# Supplementary material for: Convergence of resistance and evolutionary responses in Escherichia coli and Salmonella enterica co-inhabiting chicken farms in China
Source: Nat Commun. 2024 Jan 5;15:206. doi: 10.1038/s41467-023-44272-1 (PMC10770378; doi:10.1038/s41467-023-44272-1)
Supplement: Supplementary file 1 — Supplementary Information [file 41467_2023_44272_MOESM1_ESM.pdf]

Supplementary Materials for

Convergence of resistance and evolutionary responses in Escherichia coli and Salmonella enterica co-inhabiting chicken farms in China

Michelle Baker *et al*

*Corresponding author. Email: tania.dottorini@nottingham.ac.uk

This PDF file includes:

- **Supplementary Figures:** Figs. S1 to S22
- **Supplementary Tables:** Tables 1 to 6
- **Supplementary Notes:** Supplementary Notes 1 to 6

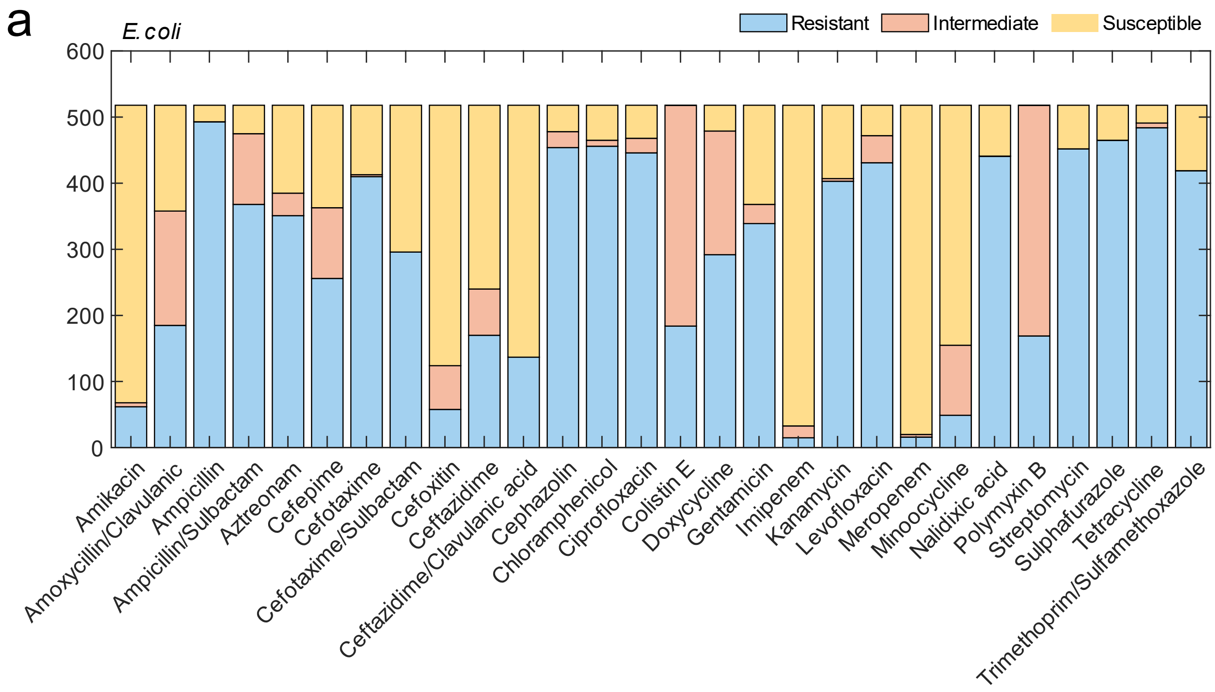

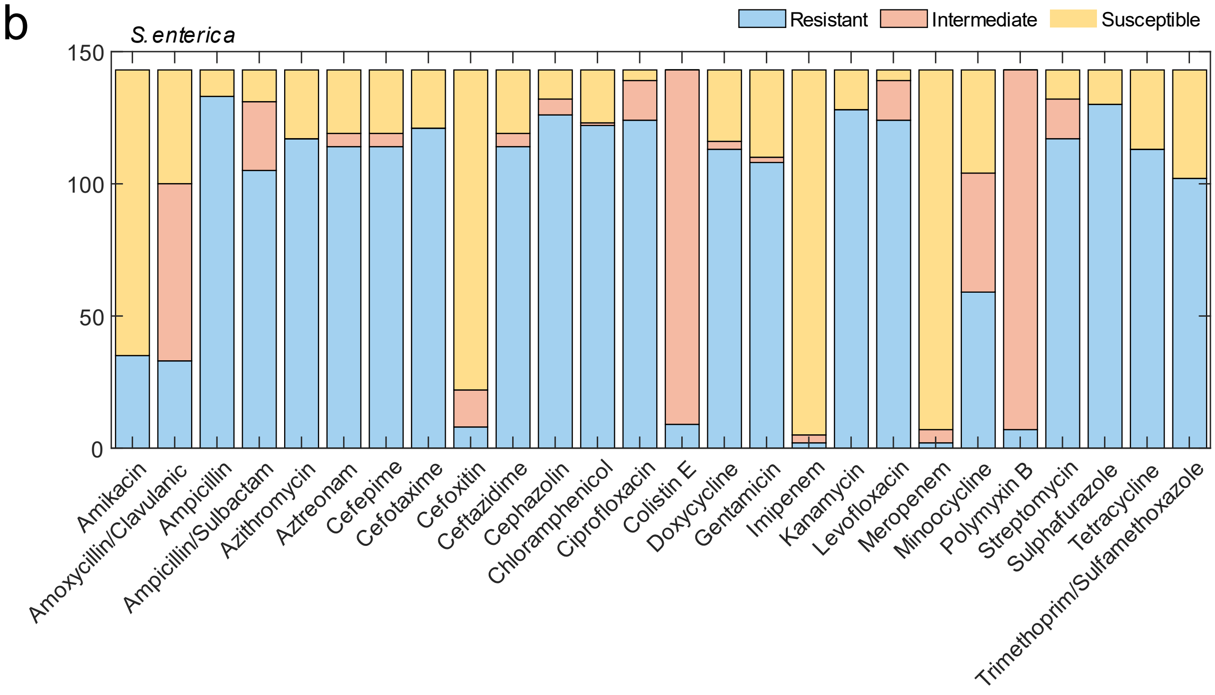

Fig. S1. Number of susceptible, intermediate, and resistant *E. coli* and *S. enterica* isolates, based on antimicrobial susceptibility testing by broth microdilution. (a) AMR phenotypes for 518 *E. coli* isolates tested against a panel of 28 antibiotics. (b) AMR phenotypes for 143 *S. enterica* isolates tested against a panel of 26 antibiotics.

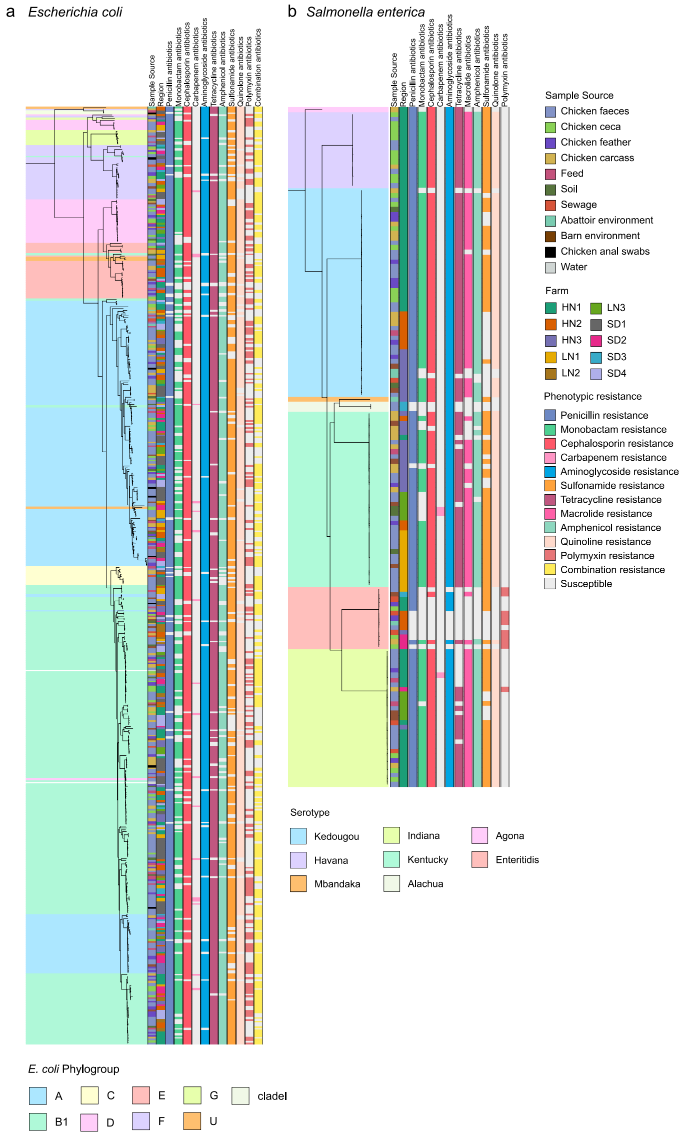

Fig. S2. Phenotypic Resistance patterns of *E. coli* and *S. enterica* isolates collected from 10 commercial broiler farms in China (a) Maximum likelihood phylogenetic tree of the whole cohort of *E. coli* isolates based on core genome of the 518 isolates, cultured from the animal and environmental samples collected from the 10 farms and 4 abattoirs. Phylogroups are shown as coloured sections. Sample source, region and phenotypic resistances are shown as strips beside the tree. (b) Maximum likelihood phylogenetic tree of the whole cohort of *S. enterica* isolates based on core genome of the 143 isolates, cultured from the animal and environmental samples collected from the 10 farms and 4 abattoirs. Serotypes are shown as coloured sections. Sample source, region and phenotypic resistances are shown as strips beside the tree. In both panels a and b the phenotypic resistance rings are grouped by antibiotic class, with presence (coloured) defined as resistance to at least one antibiotic in the class and absence (grey) as susceptibility to all tested antibiotics in the class. Farm names are abbreviated: Henan 1 (HN1), Henan 2 (HN2) Henan 3 (HN3), Liaoning 1 (LN1), Liaoning 2 (LN2), Liaoning 3 (LN3), Shandong 1 (SD1), Shandong 2 (SD2), Shandong 3 (SD3), Shandong 4 (SD4).

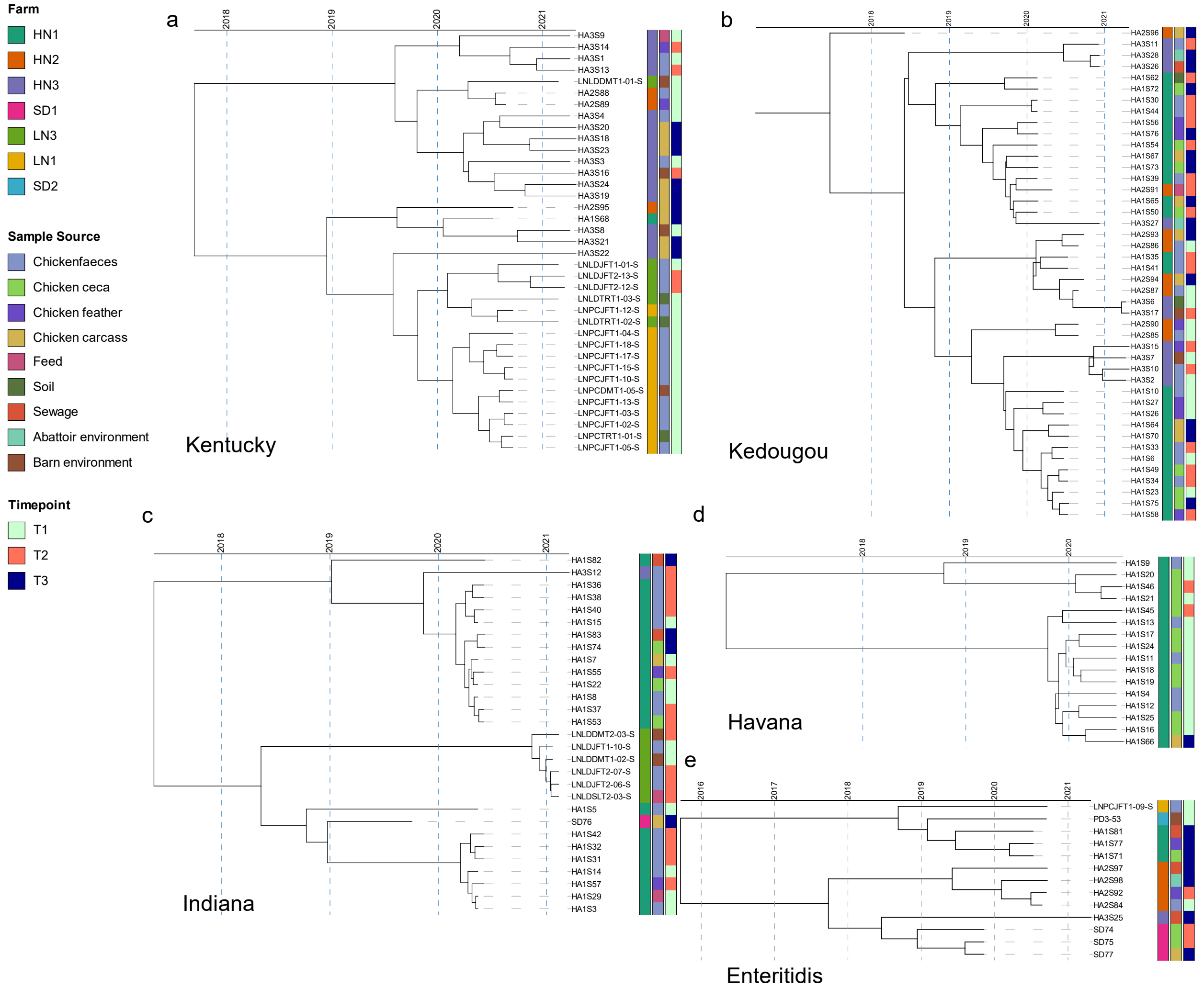

Fig S3. Bayesian divergence analysis of *S. enterica* isolates in five serotypes (a) Kentucky; (b) Kedougou; (c) Indiana; (d) Havana and (e) Enteritidis. The sample source and the farm the isolates were taken from and the timepoint for each sample are displayed as coloured strips. Farm names are abbreviated: Henan 1 (HN1), Henan 2 (HN2) Henan 3 (HN3), Liaoning 1 (LN1), Liaoning 2 (LN2), Liaoning 3 (LN3), Shandong 1 (SD1), Shandong 2 (SD2), Shandong 3 (SD3), Shandong 4 (SD4). Timepoints are abbreviated timepoint 1 (T1), timepoint 2 (T2) and timepoint 3 (T3).

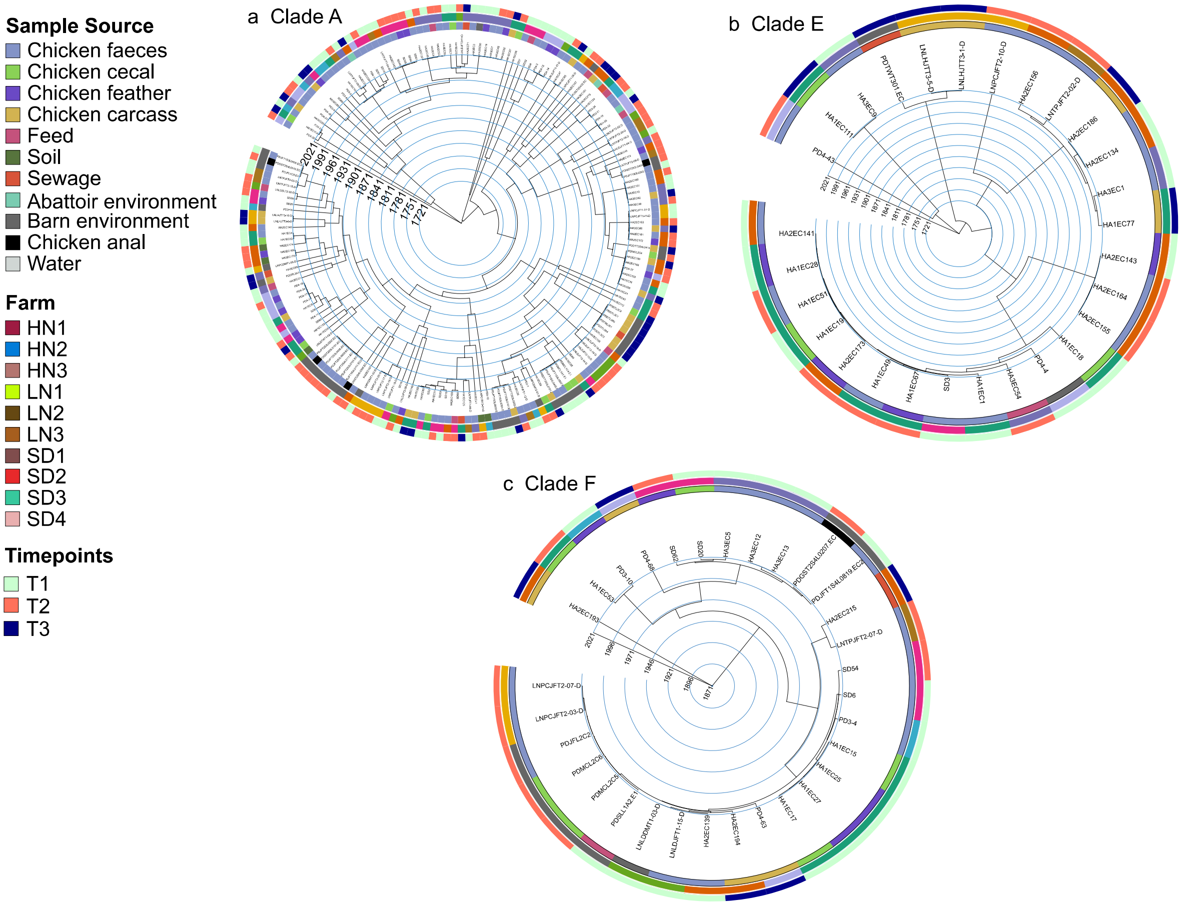

Fig S4. Bayesian divergence analysis of *E. coli* isolates in five phylogroups (a) Clade A; (b) Clade E and (c) Clade F. Sample source and the farm the isolates were taken from and the timepoint for each sample are displayed as coloured rings. Farm names are abbreviated: Henan 1 (HN1), Henan 2 (HN2) Henan 3 (HN3), Liaoning 1 (LN1), Liaoning 2 (LN2), Liaoning 3 (LN3), Shandong 1 (SD1), Shandong 2 (SD2), Shandong 3 (SD3), Shandong 4 (SD4). Timepoints are abbreviated: timepoint 1 (T1), timepoint 2 (T2) and timepoint 3 (T3).

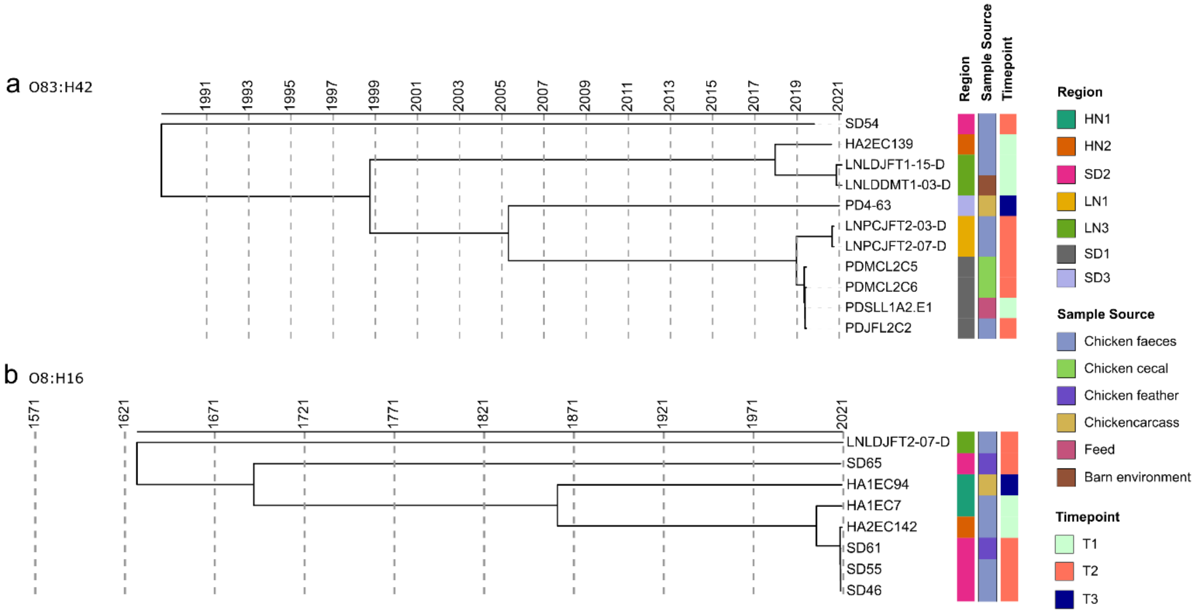

Fig S5. Bayesian divergence analysis of *E. coli* isolates in two serotypes (a) O83:H42; (b) O8:H16. the farm the isolates were taken from, sample source, and the timepoint for each sample are displayed as coloured strips. Farm names are abbreviated: Henan 1 (HN1), Henan 2 (HN2), Liaoning 1 (LN1), Liaoning 3 (LN3), Shandong 1 (SD1), Shandong 2 (SD2), Shandong 3 (SD3). Timepoints are abbreviated: timepoint 1 (T1), timepoint 2 (T2) and timepoint 3 (T3).

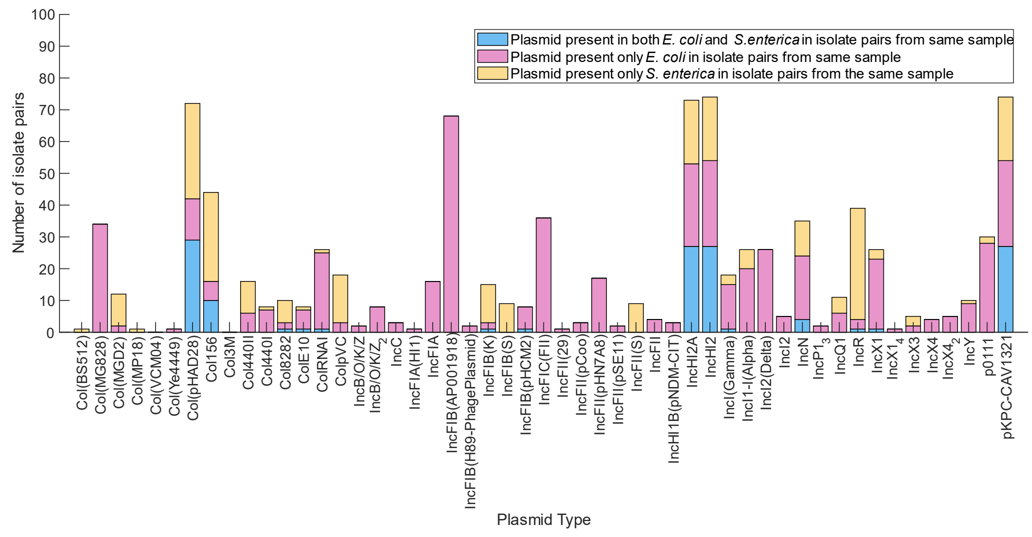

Fig S6. Plasmid presence in *E. coli* and *S. enterica* isolate pairs from the same samples. Number of isolate pairs with the same plasmids in both *E. coli* and *S. enterica* (blue), *E. coli* only (pink) and *S. enterica* only (yellow), separated by replicon type. Only isolates where both species were isolated from the same sample were included in this analysis (i.e. 113 *E. coli* and 113 *S. enterica* isolates).

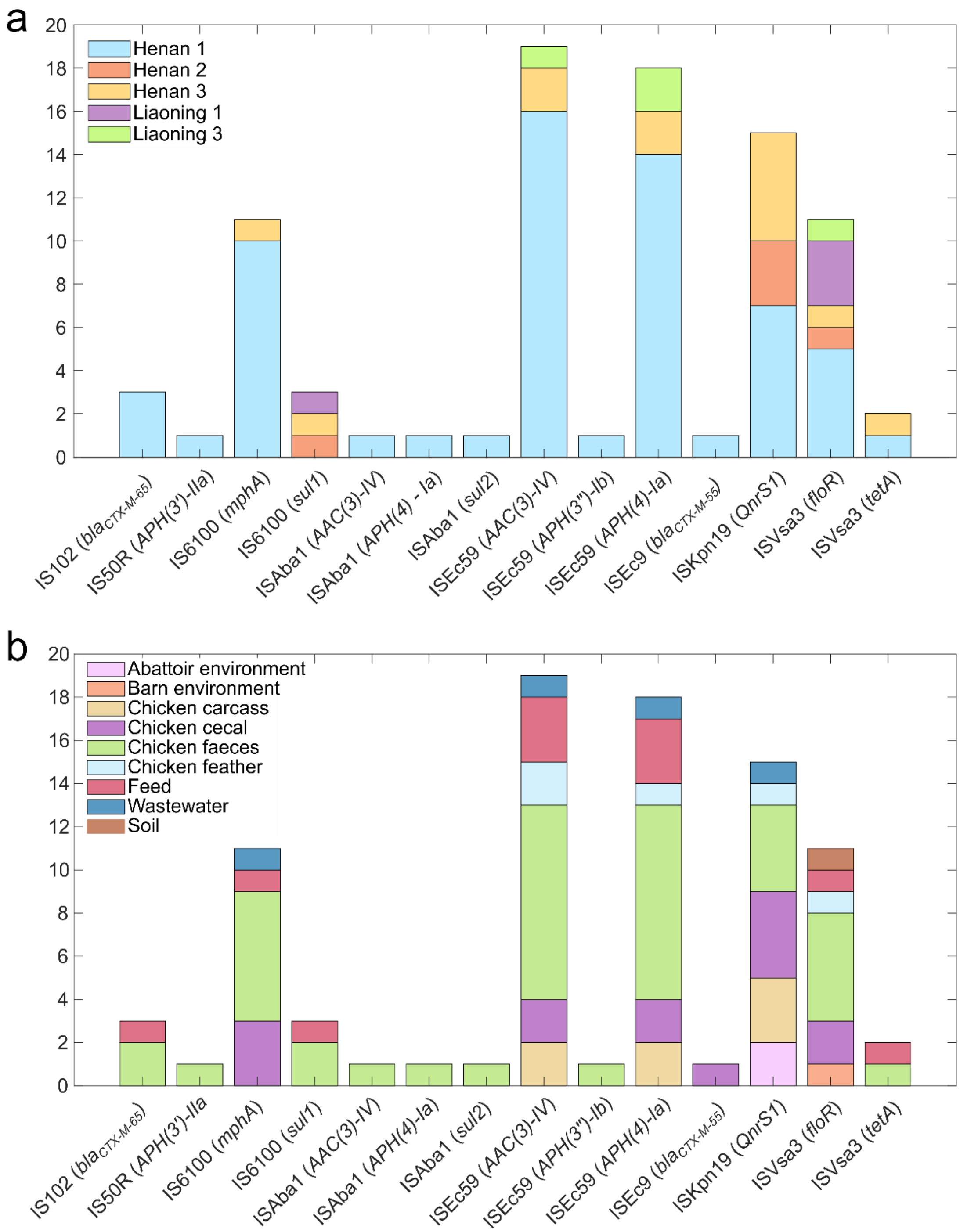

Fig S7. Numbers of different mobile ARGs found in isolate pairs of *S. enterica* and *E. coli* taken from the same samples. Of 113 *E. coli* and *S. enterica* isolates pairs collected from the same samples, 88 were found to have the same mobile ARG. (a) Stacked bar plot showing the breakdown, by farm, of mobile ARGs found in both *E. coli* and *S. enterica* isolates. (b) Stacked bar plot showing the breakdown by source type of mobile ARGs found in both *E. coli* and *S. enterica* isolates.

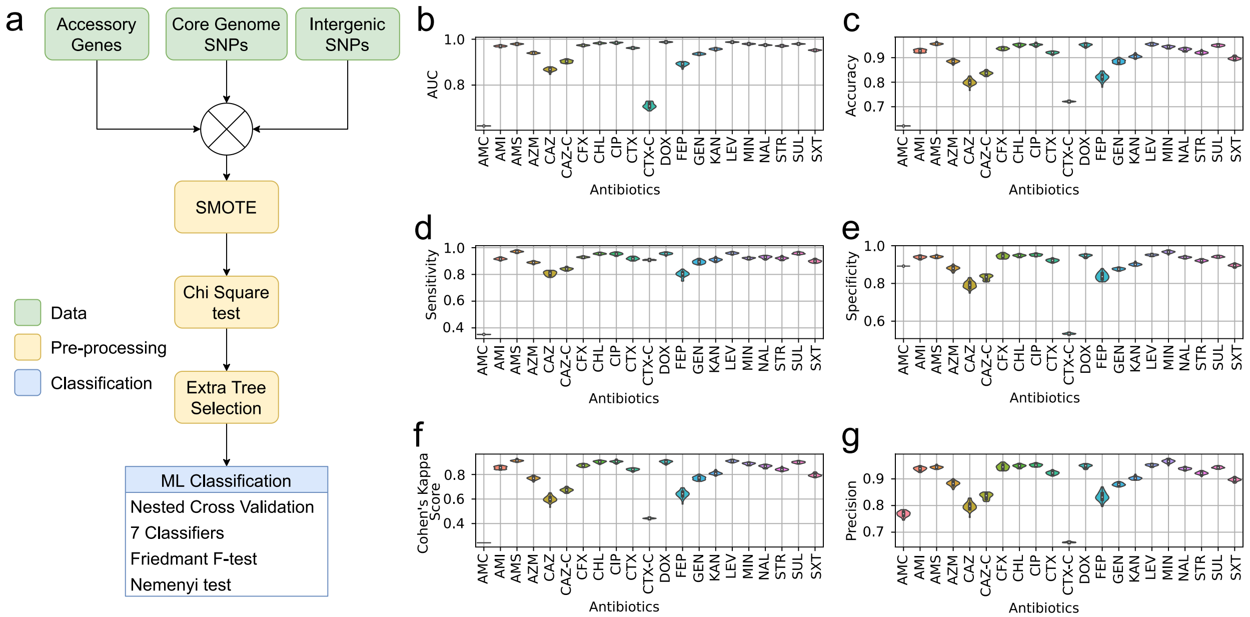

Fig S8. Supervised machine learning pipeline accurately predicts *E. coli* resistance susceptibility profiles. (a) Flow diagram showing machine learning pipeline including data (green), pre-processing steps (yellow) and classification (blue). (b) – (g) Machine learning performance results for six performance indicators: (b) area under the curve AUC, (c) accuracy, (d) sensitivity, (e) Specificity, (f) Cohen's kappa score, and (g) precision from 30 training runs for each antimicrobial. The results shown are for the best classifier Random Forest, as defined by the Nemenyi test (Fig. S10a). Predictive models were generated for twenty-one different antimicrobials (X axis): amoxycillin/clavulanic acid (AMC), amikacin (AMI), ampicillin/sulbactam (AMS), aztreonam (AZM), ceftazidime (CAZ), ceftazidime/clavulanic acid (CAZ-C), cefoxitin (CFX), chloramphenicol (CHL), ciprofloxacin (CIP), cefotaxime (CTX), cefotaxime/ clavulanic acid (CTX-C), doxycycline (DOX), cefepime (FEP), gentamicin (GEN), kanamycin (KAN), levofloxacin (LEV), minocycline (MIN), nalidixic acid (NAL), streptomycin (STR), sulfisoxazole (SUL), and trimethoprim/sulfamethoxazole (SXT).

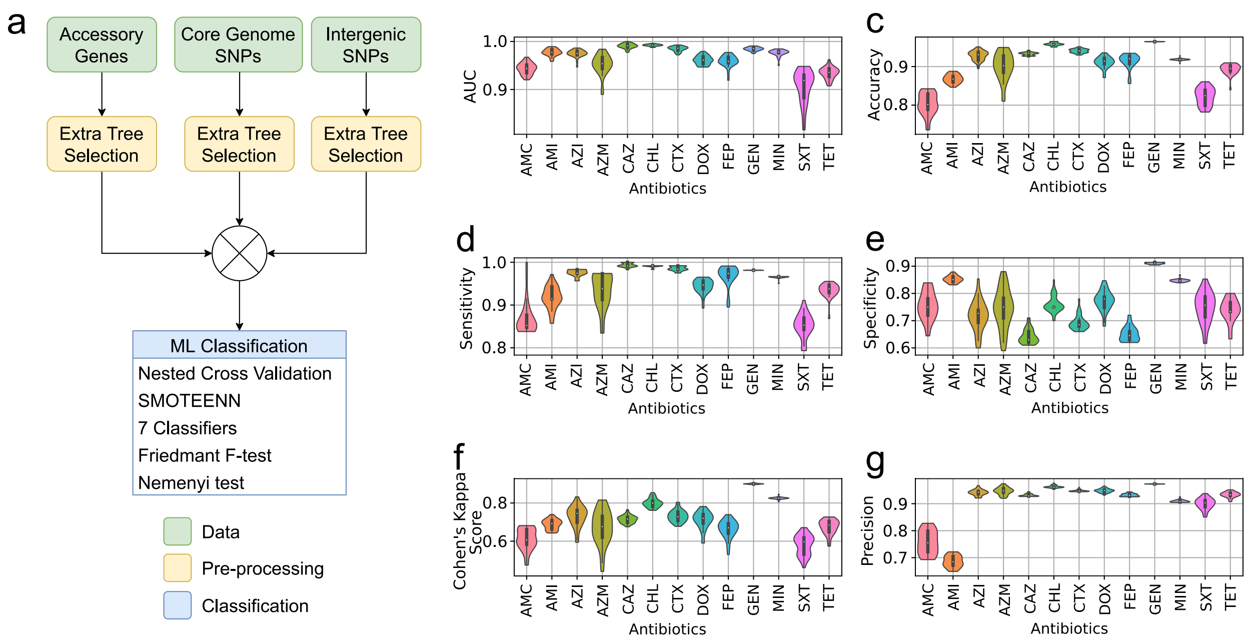

Figure S9. Supervised machine learning pipeline accurately predicts *S. enterica* resistance susceptibility profiles. (a) Flow diagram showing machine learning pipeline including data (green), pre-processing steps (yellow) and classification (blue). (b) – (g) Machine learning performance results for six performance indicators: (b) area under the curve AUC, (c) accuracy, (d) sensitivity, (e) Specificity, (f) Cohen's kappa score, and (g) precision from 30 training runs for each antimicrobial. The results shown are for the best classifier Linear SVM, as defined by the Nemenyi test (Fig. S10b). Predictive models were generated for thirteen different antimicrobials (X axis): amoxycillin/clavulanic acid (AMC), amikacin (AMI), azithromycin (AZI), aztreonam (AZM), ceftazidime (CAZ), chloramphenicol (CHL), cefotaxime (CTX), doxycycline (DOX), cefepime (FEP), gentamicin (GEN), minocycline (MIN), trimethoprim/sulfamethoxazole (SXT), and tetracycline (TET).

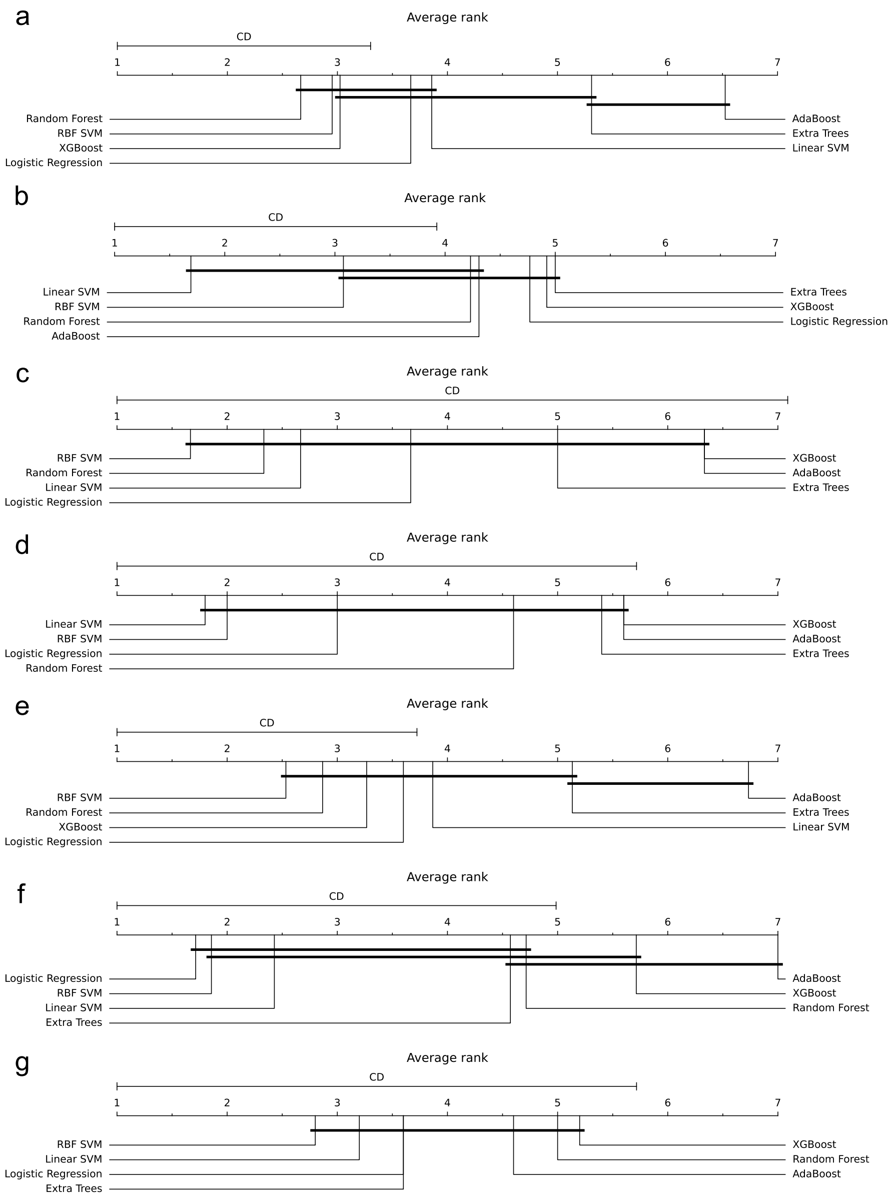

Fig S10. Nemenyi *post-hoc* tests. Comparison of the performance of the 5 classifiers and 2 meta-methods, using their average ordinal rank over the antibiotics analysed based on the AUC performance metric for (a) *E. coli* Chinese all isolates, (b) *S. enterica* Chinese all isolates, (c) *E. coli* Chinese co-inhabiting chicken isolates, (d) *S. enterica* Chinese co-inhabiting Chicken isolates, (e) *E. coli* Chinese not co-inhabiting chicken isolates, (f) *E. coli* EFFORT not-necessarily co-inhabiting chicken isolates and (g) *S. enterica* ENGAGE not-necessarily co-inhabiting chicken isolates. The x-axis indicates the average ordinal rank of the machine learning methods. The scale is from 1 (best rank) to 7 (worst rank). The ordinal rank of a classifier is defined as follows: the ML method with the best AUC is given rank 1, the second-best AUC rank 2 and the *n*-th AUC best rank *n*, with *n* being the number of machine learning methods used. For each antibiotic, the methods are ranked between 1 (highest AUC) and 7 (lowest AUC), since in this case there are 7 machine learning methods used. Next, for each method, the ranks are averaged based on the 11 antibiotics studied. The critical distance (CD) is defined based on the Nemenyi *post-hoc* test, all the methods that fall in the same bold bar below the axis are considered statistically equivalent based on the CD value .

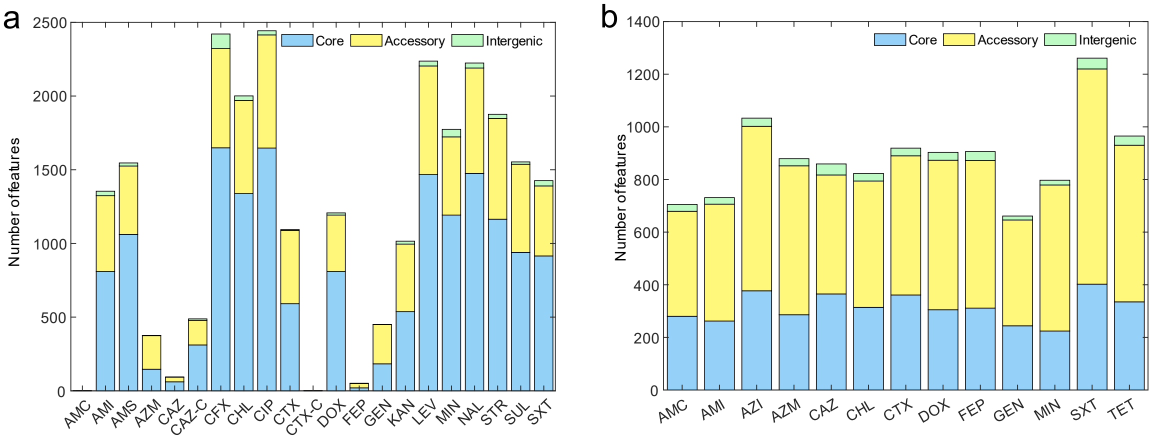

Fig S11. Number of features correlated to resistance-susceptibility profiles by ML. For each antibiotic model for (a) *E. coli* and (b) *S. enterica*, the number of core (blue), accessory (yellow) and intergenic (green) features are shown. The antibiotics shown are amoxicillin/clavulanic acid (AMC), amikacin (AMI), ampicillin/sulbactam (AMS), azithromycin (AZI), aztreonam (AZM), ceftazidime (CAZ), ceftazidime/clavulanic acid (CAZ-C), cefoxitin (CFX), chloramphenicol (CHL), ciprofloxacin (CIP), cefotaxime (CTX), cefotaxime/ clavulanic acid (CTX-C), doxycycline (DOX), cefepime (FEP), gentamicin (GEN), kanamycin (KAN), levofloxacin (LEV), minocycline (MIN), nalidixic acid (NAL), streptomycin (STR), sulfisoxazole (SUL), trimethoprim/sulfamethoxazole (SXT), and tetracycline (TET).

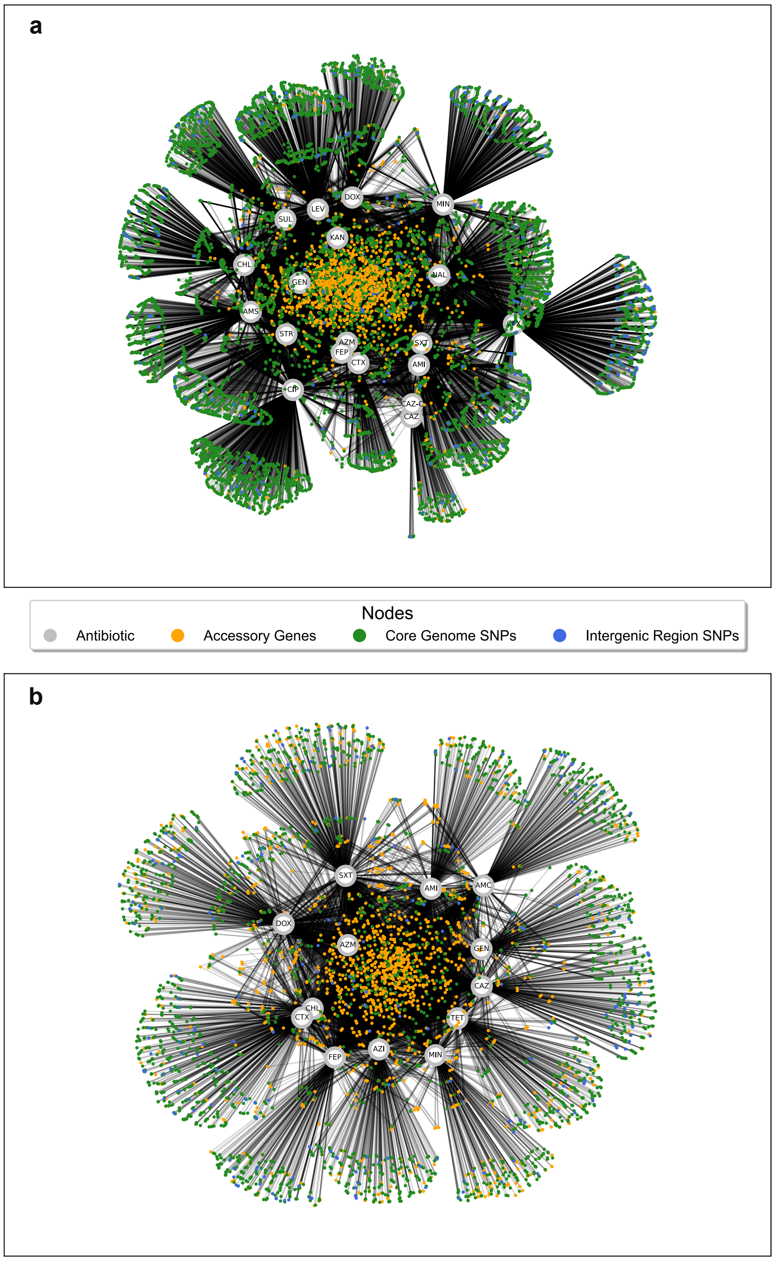

Fig S12. Undirected graph network indicating the genomic features found for each antibiotic model for (a) *E. coli* and (b) *S. enterica*. The colour of the node indicates which genomic feature it belongs to accessory genes are in orange, core genome SNPs in green and intergenic region SNPs in blue. The antibiotic models are coloured in grey, and indicated as three letter antibiotic abbreviations: amoxycillin/clavulanic acid (AMC), amikacin (AMI), ampicillin/sulbactam (AMS), azithromycin (AZI), aztreonam (AZM), ceftazidime (CAZ), ceftazidime/clavulanic acid (CAZ-C), cefoxitin (CFX), chloramphenicol (CIP), ciprofloxacin (CIP), cefotaxime (CTX), cefotaxime/ clavulanic acid (CTX-C), doxycycline (DOX), cefepime (FEP), gentamicin (GEN), kanamycin (KAN), levofloxacin (LEV), minocycline (MIN), nalidixic acid (NAL), streptomycin (STR), sulfisoxazole (SUL), trimethoprim/sulfamethoxazole (SXT) and tetracycline (TET).

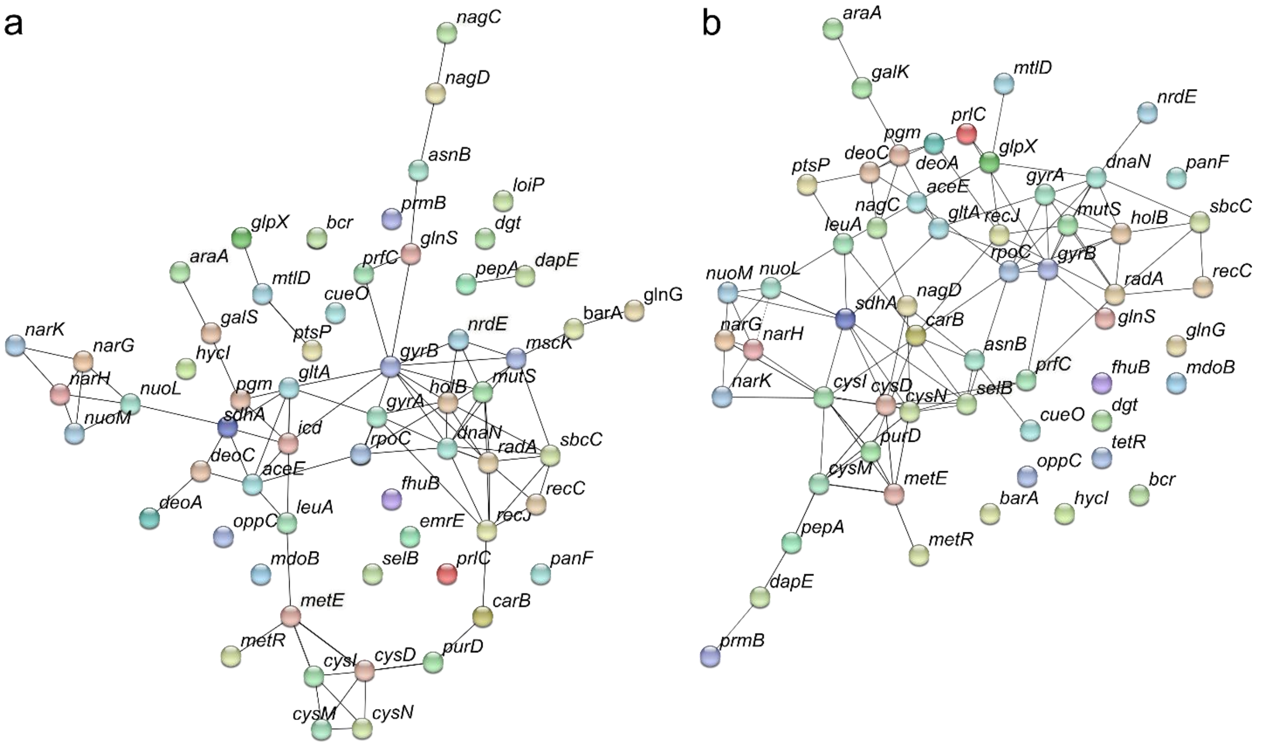

Figure S13. The most significant genes correlated to AMR profiles, as predicted by ML, in both species show high levels of protein-protein interaction. Genes that were in the top 10% of features (ranked according to the maximum feature importance in 30 runs) in both species, *E. coli* and *S. enterica* are plotted in a protein-protein interaction network, according String database interactions (confidence threshold 0.4). (a) genes plotted according to interactions in *E. coli K12 MG1655*. (b) genes plotted according to interactions in *S. enterica subsp. enterica serovar Typhimurium str. LT2*. Network generated by StringDB v11.5.

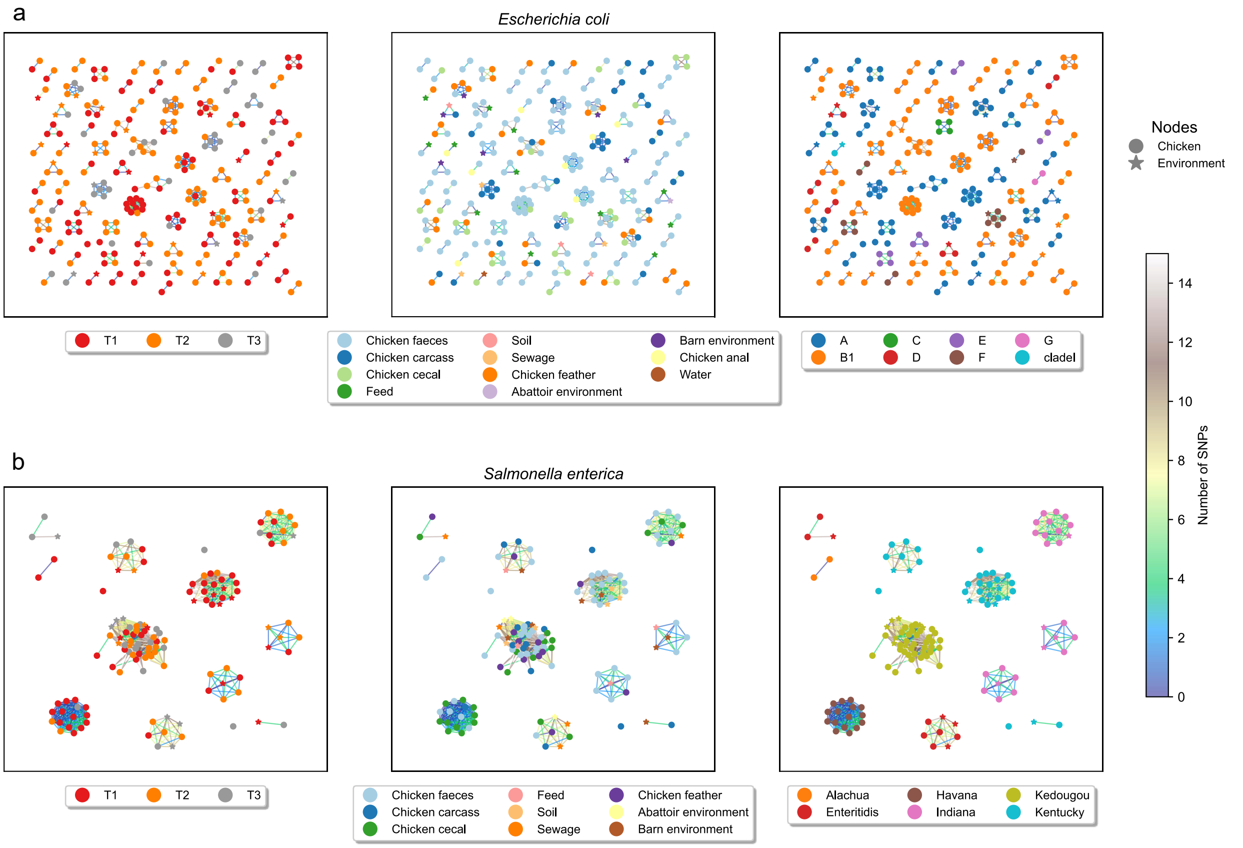

Fig. S14. SNP network analysis of highly connected isolates in *S. enterica* and *E. coli*. Network diagram showing pairwise connections between chicken (circle) and environmental (star) isolates with less than 15 pairwise SNP differences for (a) *S. enterica* and (b) *E. coli*. The panels in each row show the same network with the nodes colour-coded according to time point (left), source type (centre) or serotype/phylogroup (right). The lines between pairs of isolates are colour-coded by SNP number. Timepoints are abbreviated: timepoint 1 (T1), timepoint 2 (T2) and timepoint 3 (T3).

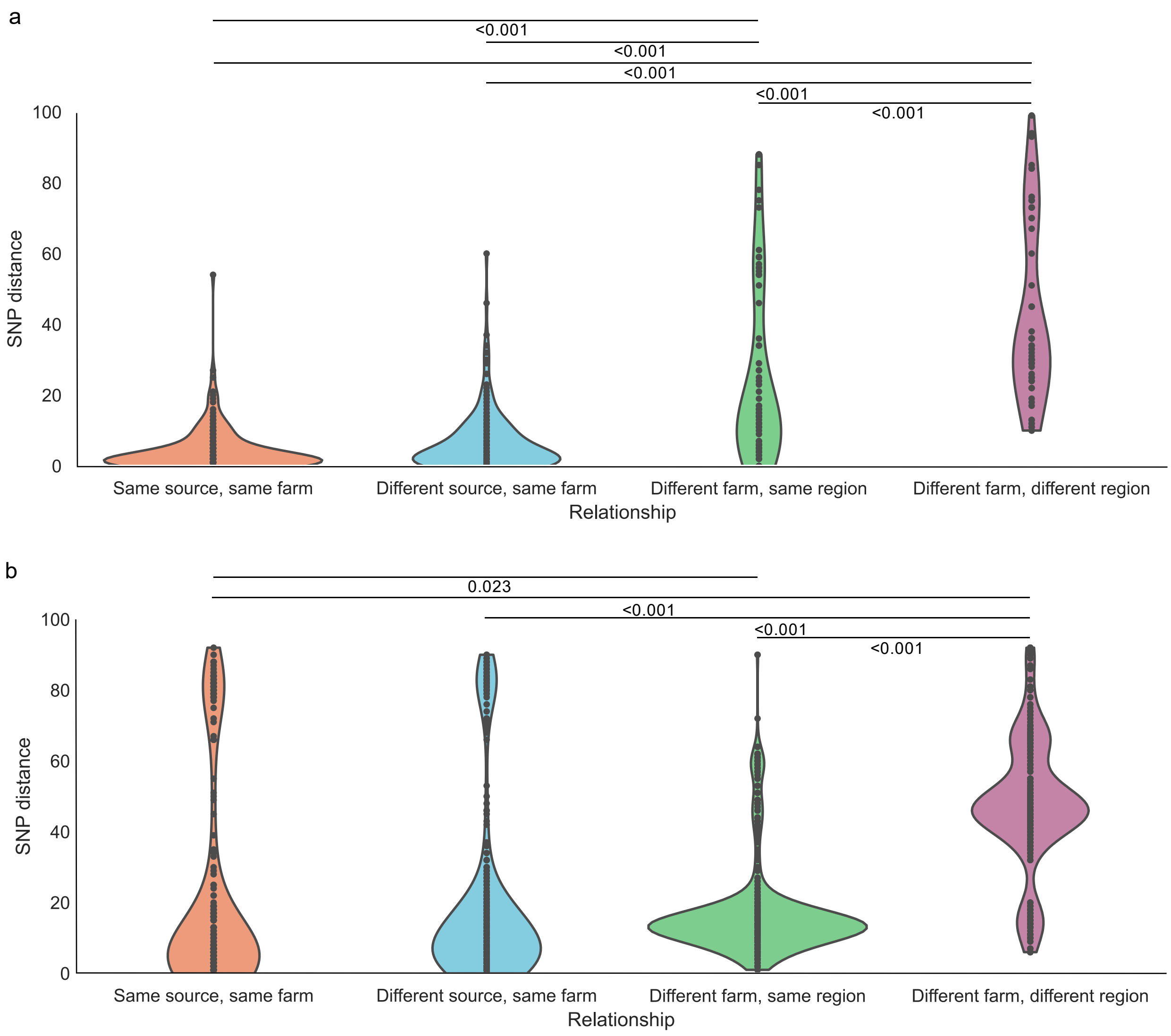

Fig. S15. Distribution of pairwise comparisons of SNP distance between (a) *E. coli* and (b) *S. enterica* isolates across source types, different farms, and provinces. Only pairs with less than 100 SNPs were included in the analysis[1]. Differences in the means were tested using a pairwise Tukey HSD test with FWER control (<0.05), with significance measured as adjusted p-values < 0.05. Adjusted p-values of significant tests are shown on the plot.

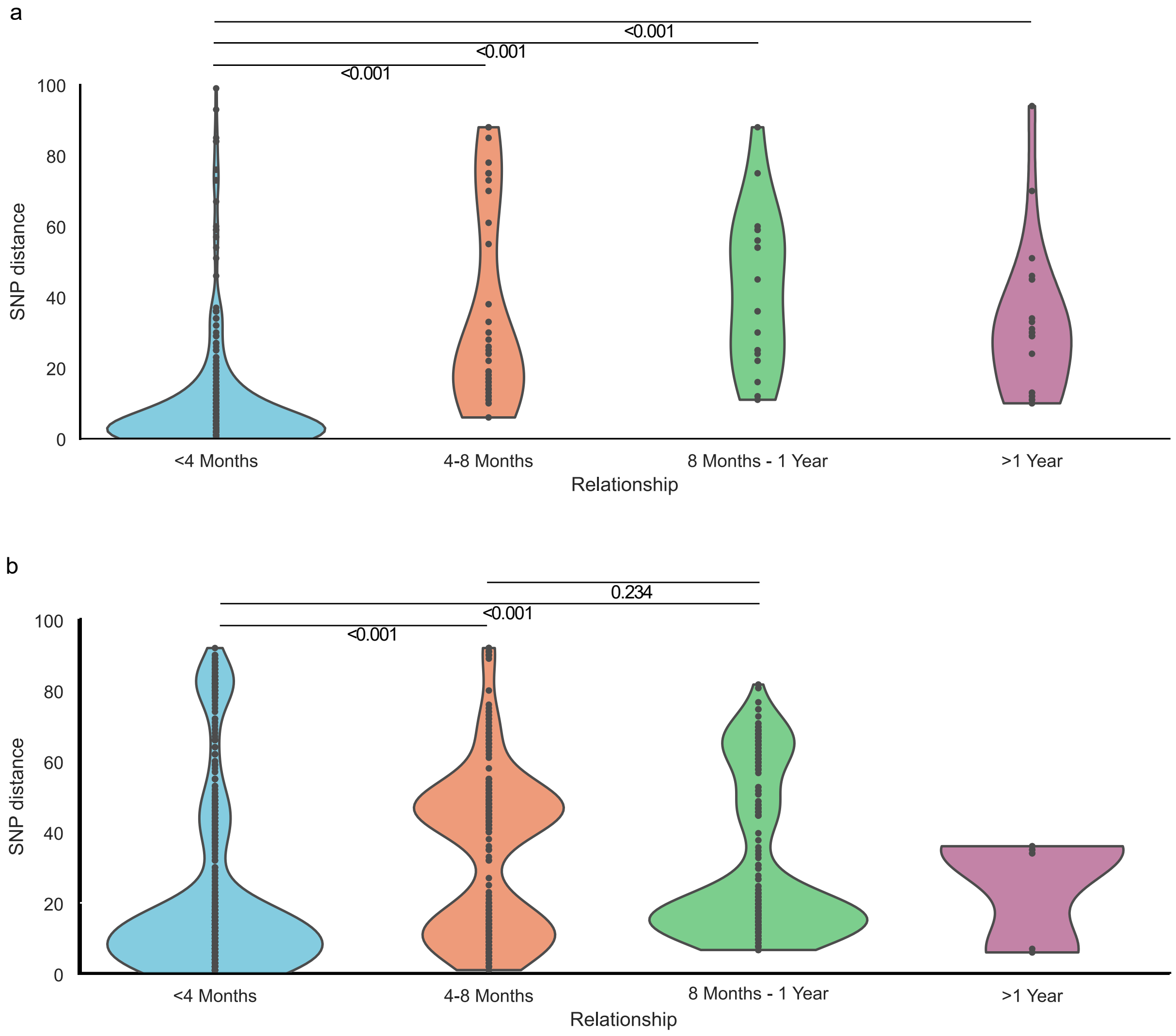

Fig. S16. Distribution of pairwise comparisons of SNP distance between (a) *E. coli* and (b) *S. enterica* isolates across collection dates. Only pairs with less than 100 SNPs were included in the analysis[1]. The distribution categories refer to the difference in dates between each isolate pair. Differences in the means were tested using a pairwise Tukey HSD test with FWER control (<0.05), with significance measured as adjusted *p*-values < 0.05. Adjusted *p*-values of significant tests are shown on the plot.

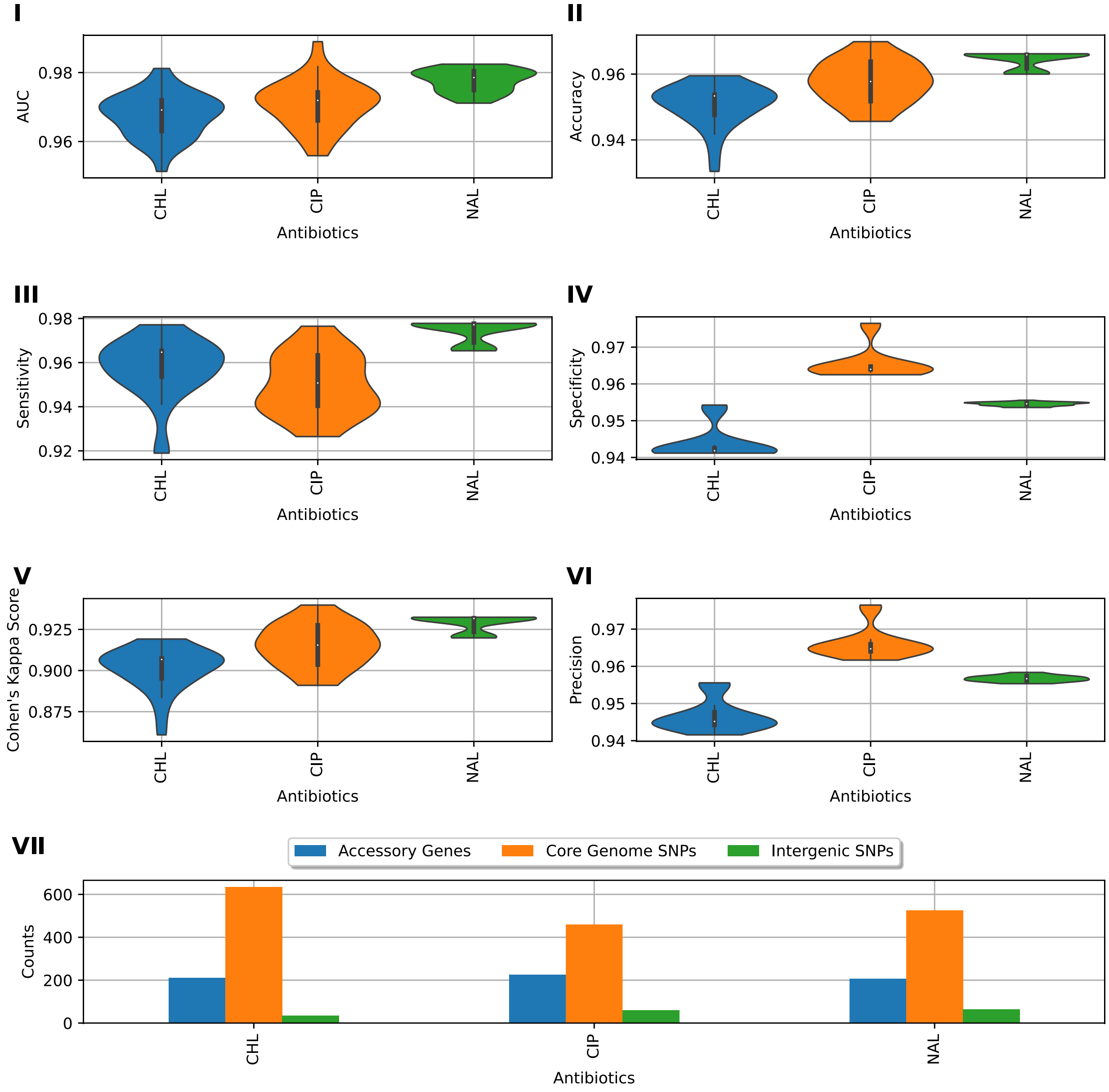

Fig. S17. The supervised machine learning pipeline effectively predicts the resistance/susceptibility profiles of co-inhabiting *E. coli* isolates. This prediction is based on a dataset comprising 98 isolates collected from a Chinese cohort and originating from various chicken sources, including faeces, ceca, carcasses, and feathers. Machine learning performance results are given for six performance indicators: (I) area under the curve AUC, (II) accuracy, (III) sensitivity, (IV) Specificity, (V) Cohen's kappa score, and (VI) precision. All indicators were calculated from 30 training runs for each antimicrobial model. (VII) Number and type of features (accessory genes, core genome SNPs and intergenic region SNPs) selected by each antibiotic model. The results shown are for the best classifier RBF-SVM, as defined by the Nemenyi test (Fig. S10c). The antibiotic models are abbreviated: chloramphenicol (CHL), ciprofloxacin (CIP), and nalidixic acid (NAL).

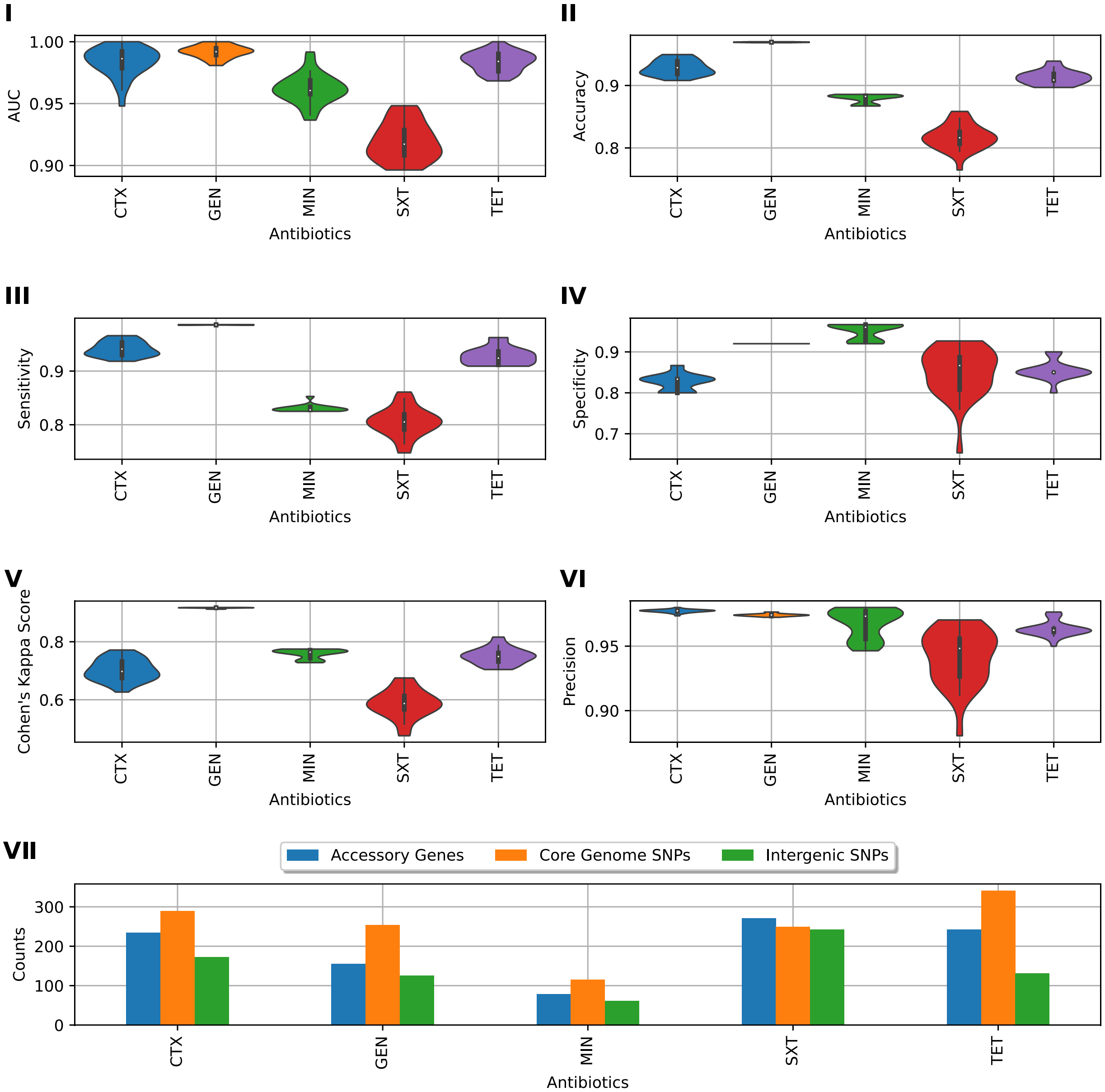

Fig. S18. The supervised machine learning pipeline effectively predicts the resistance/susceptibility profiles of co-inhabiting *S. enterica* isolates. This prediction is based on a dataset comprising 98 isolates collected from a Chinese cohort and originating from various chicken sources, including faeces, ceca, carcasses, and feathers. Machine learning performance results are given for six performance indicators: (I) area under the curve AUC, (II) accuracy, (III) sensitivity, (IV) Specificity, (V) Cohen's kappa score, and (VI) precision. All indicators were calculated from 30 training runs for each antimicrobial model. (VII) Number and type of features (accessory genes, core genome SNPs and intergenic region SNPs) selected by each antibiotic model. The results shown are for the best classifier Linear SVM, as defined by the Nemenyi test (Fig. S10d). The antibiotic models are abbreviated: cefotaxime (CTX), cefotaxime/ clavulanic acid (CTX-C), gentamicin (GEN), minocycline (MIN), trimethoprim/sulfamethoxazole (SXT) and tetracycline (TET).

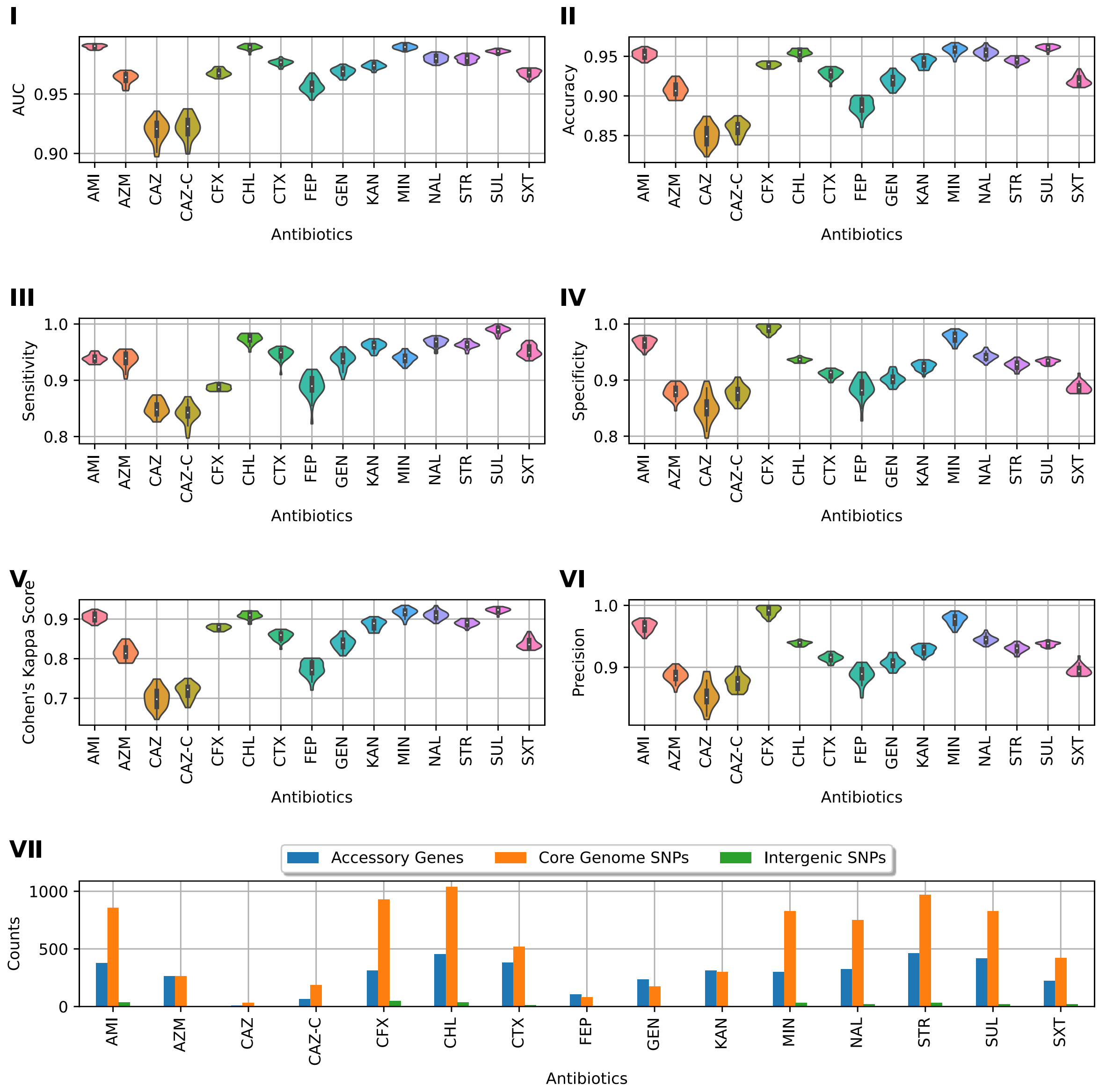

Fig. S19. The machine learning pipeline effectively predicts the resistance/susceptibility profiles of Chinese not co-inhabiting *E. coli* isolates. This prediction is based on a dataset comprising 337 isolates collected from a Chinese cohort and originating from various chicken sources, including faeces, ceca, carcasses, and feathers. Machine learning performance results are given for six performance indicators: (I) area under the curve AUC, (II) accuracy, (III) sensitivity, (IV) Specificity, (V) Cohen's kappa score, and (VI) precision. All indicators were calculated from 30 training runs for each antimicrobial model. (VII) Number and type of features (accessory genes, core genome SNPs and intergenic region SNPs) selected by each antibiotic model. The results shown are for the best classifier RBF-SVM, as defined by the Nemenyi test (Fig. S10e). The antibiotic models are abbreviated: amikacin (AMI), aztreonam (AZM), ceftazidime (CAZ), ceftazidime/clavulanic acid (CAZ-C), cefoxitin (CFX), chloramphenicol (CHL), cefotaxime (CTX), cefepime (FEP), gentamicin (GEN), kanamycin (KAN), minocycline (MIN), nalidixic acid (NAL), streptomycin (STR), sulfisoxazole (SUL) and trimethoprim/sulfamethoxazole (SXT).

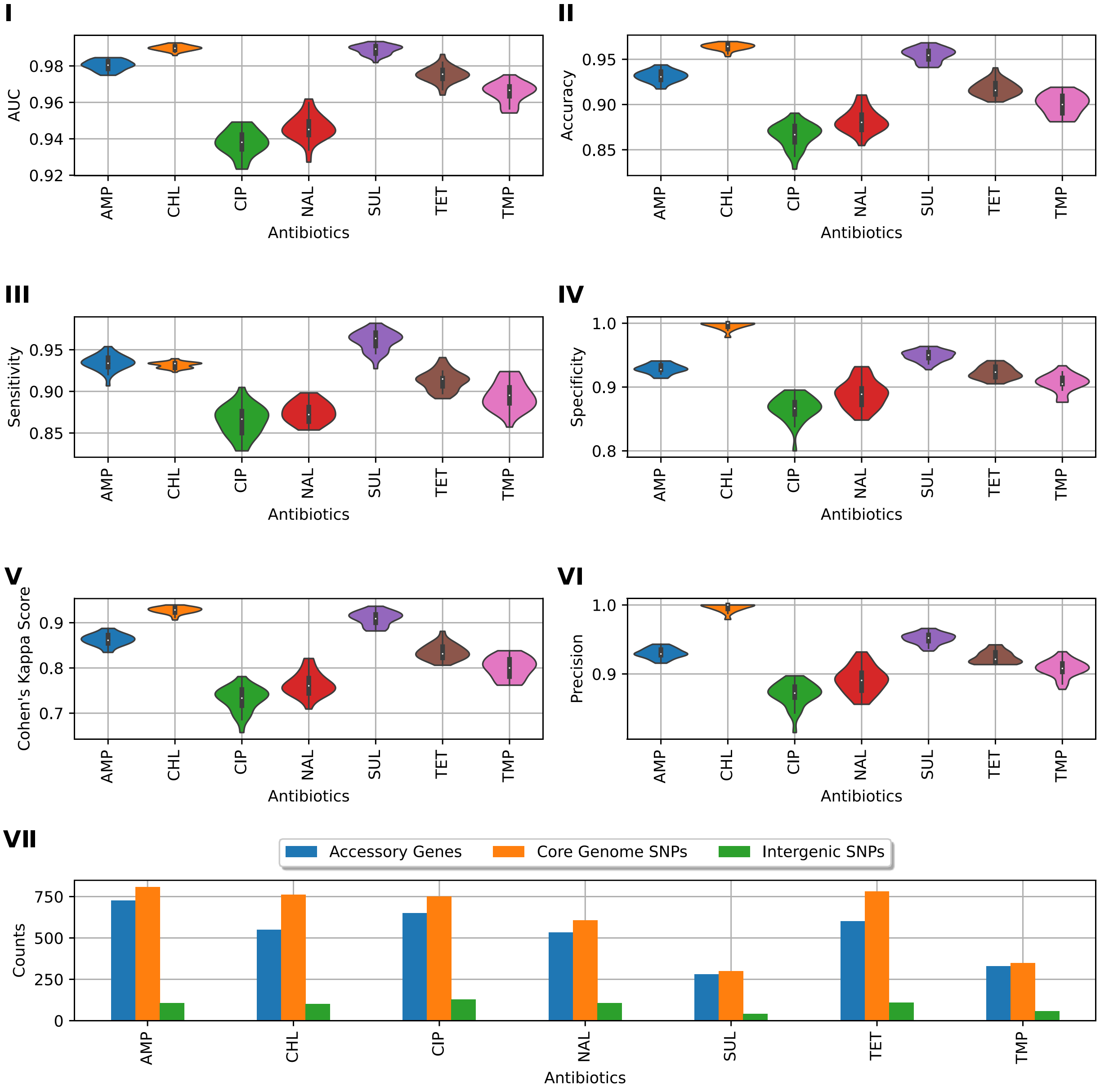

Fig. S20. The supervised machine learning pipeline effectively predicts the resistance/susceptibility profiles of EFFORT *E. coli* isolates. This prediction is based on a dataset comprising 206 isolates collected from a European cohort and originating from chicken faeces, with strong likelihood of absence of co-inhabiting *S. enterica*. Machine learning performance results are given for six performance indicators: (I) area under the curve AUC, (II) accuracy, (III) sensitivity, (IV) Specificity, (V) Cohen's kappa score, and (VI) precision. All indicators were calculated from 30 training runs for each antimicrobial model. (VII) Number and type of features (accessory genes, core genome SNPs and intergenic region SNPs) selected by each antibiotic model. The results shown are for the best classifier Logistic Regression, as defined by the Nemenyi test (Fig. S10f). The antibiotic models are abbreviated: ampicillin (AMP), chloramphenicol (CHL), ciprofloxacin (CIP), nalidixic acid (NAL), sulfisoxazole (SUL), trimethoprim/sulfamethoxazole (TMP) and tetracycline (TET).

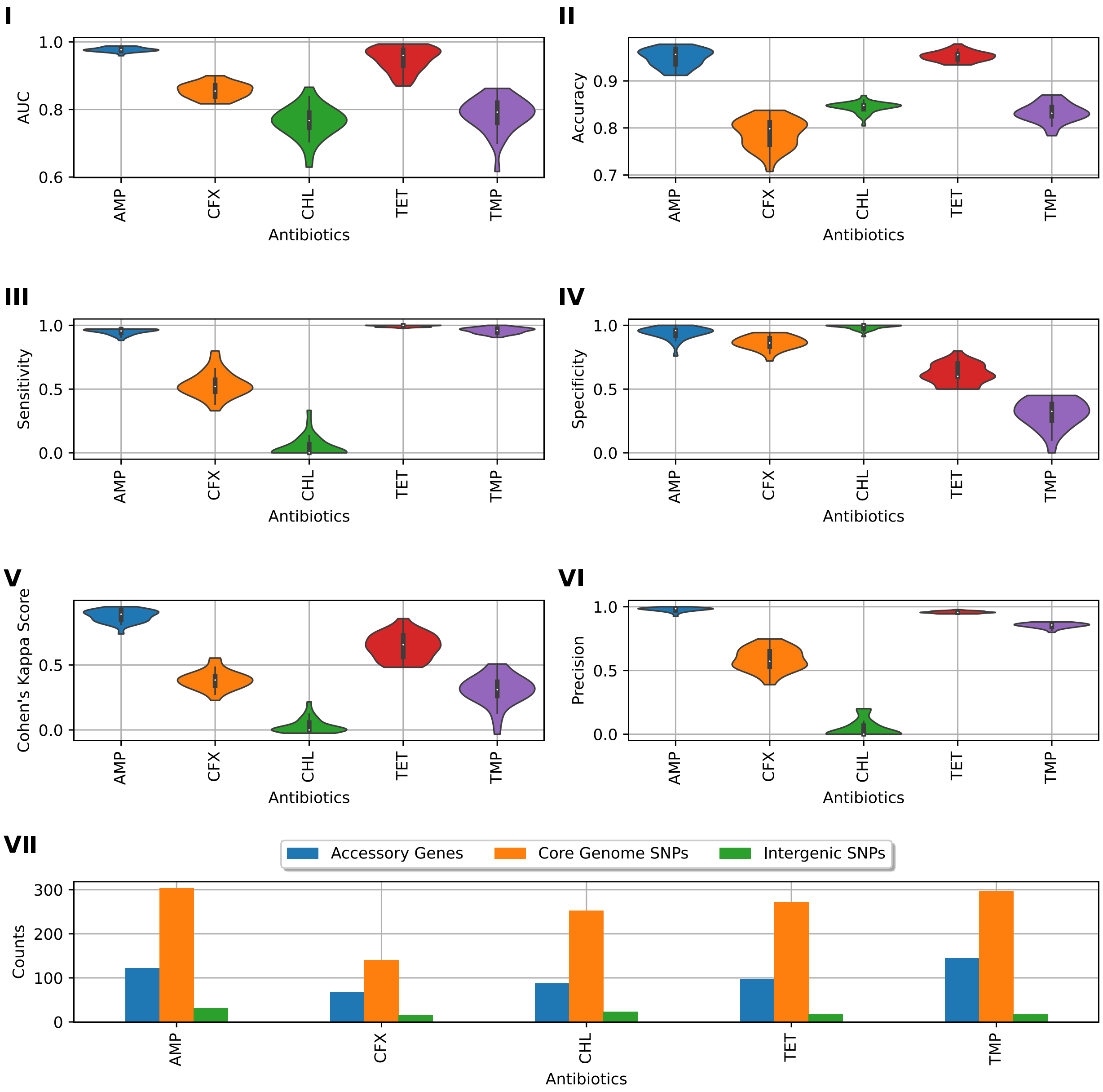

Fig. S21. The supervised machine learning pipeline effectively predicts the resistance/susceptibility profiles of ENGAGE *S. enterica* isolates (from samples which may or may not contain also co-inhabiting *E. coli*). The prediction is based on a dataset comprising 92 isolates collected from an Italian cohort and originating from chicken faeces and cecum. Machine learning performance results are given for six performance indicators: (I) area under the curve AUC, (II) accuracy, (III) sensitivity, (IV) Specificity, (V) Cohen's kappa score, and (VI) precision. All indicators were calculated from 30 training runs for each antimicrobial model. (VII) Number and type of features (accessory genes, core genome SNPs and intergenic region SNPs) selected by each antibiotic model. The results shown are for the best classifier RBF-SVM, as defined by the Nemenyi test (Fig. S10g). The antibiotic models are abbreviated: ampicillin (AMP), cefoxitin (CFX), chloramphenicol (CHL), trimethoprim/sulfamethoxazole (TMP) and tetracycline (TET).

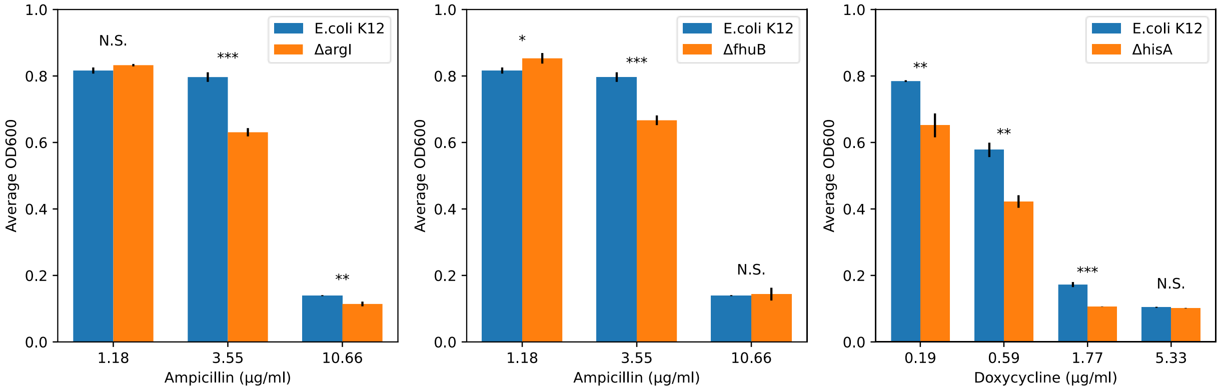

Fig. S22. Susceptibility against antimicrobials of selected mutants with knocked-down genes vs wild type. *E. coli* strains (*E. coli* K12, Δ*argI*, Δ*fhuB* and Δ*hisA* mutants) were inoculated in LB in 96-well plates amended with ampicillin or doxycycline at the indicated concentrations and incubated for 20 h at 35°C without shaking, after which the optical density at 600 nm (OD600) was measured. Significance of *t*-tests: *** $p \leq 0.001$; ** $0{,}001 \leq p \leq 0.01$; * $0.01 \leq p \leq 0.05$; N.S not significant, two-sided t-test.

Supplementary Table 1.
Bayes factors of BETS analysis for the major serotypes and phylogroups in the *E. coli* and *S. enterica* cohorts.

Species	Group	Log Bayes Factor
S. enterica	Enteritidis	1.35
S. enterica	Indiana	2.33
S. enterica	Kentucky	8.30
S. enterica	Kedougou	21.91
S. enterica	Havana	107.63
E. coli	Clade A	2759.45
E. coli	Clade B1	-919.95
E. coli	Clade D	-302.27
E. coli	Clade E	41.98
E. coli	Clade F	770.73
E. coli	O8H16	-39.406
E. coli	O83H42	-3.801

Supplementary Table 2.
Number of AMR-associated accessory genes, core genome SNPs, and core genome genes found by the machine learning pipeline, in the Chinese co-inhabiting *E. coli* and *S. enterica* isolates, with hypothetical genes excluded.

Dataset	Antibiotics	Accessory genes	Core genome SNPs	Core genome Genes
Chinese co-inhabiting *E. coli*	CHL	46	618	431
	CIP	50	443	324
	NAL	38	502	360
Chinese co-inhabiting *S. enterica*	CTX	41	257	227
	GEN	26	220	205
	MIN	18	89	87
	SXT	42	210	185
	TET	42	290	255

Supplementary Table 3.
Number of AMR-associated accessory genes, core genome SNPs, and core genome genes found by the machine learning pipeline, in the Chinese not co-inhabiting datasets for *E. coli*, with hypothetical genes excluded.

Dataset	Antibiotics	Accessory genes	Core genome SNPs	Core genome Genes

Chinese not co-inhabiting *E. coli*	AMI	94	832	478
	AZM	70	255	163
	CAZ	4	32	30
	CAZ-C	24	183	118
	CFX	70	902	500
	CHL	98	1007	574
	CTX	83	510	331
	FEP	31	81	55
	GEN	43	173	128
	KAN	64	295	209
	MIN	87	802	488
	NAL	81	730	461
	STR	110	946	547
	SUL	101	809	467
	SXT	56	411	249

Supplementary Table 4.
Percentages of AMR-associated accessory genes, core genome genes, and overall number of genes identified by machine learning in both the Chinese co-inhabiting and not co-inhabiting *E. coli* isolates. The co-inhabiting set is taken as reference. Hypothetical genes were excluded, each column gives the percentages of genes in common between the sets.

Datasets	Antibiotics	Accessory genes	Core genome genes	Overall number of genes
Not co-inhabiting Chinese *E. coli*	CHL	8.70%	43.39%	40.04%
	NAL	5.26%	42.22%	38.69%

Supplementary Table 5.
Number of AMR-associated accessory genes, core genome SNPs, and core genome genes found by the machine learning, in the European EFFORT and ENGAGE datasets, with hypothetical genes excluded.

Dataset	Antibiotics	Accessory genes	Core genome SNPs	Core genome Genes
E. coli (EFFORT)	AMP	100	744	378
	CHL	105	685	368
	CIP	98	672	284
	NAL	83	533	277
	SUL	39	272	154
	TET	87	719	335
	TMP	59	321	177
S. enterica (ENGAGE)	AMP	21	266	106
	CHL	18	217	81
	CTX	18	118	71
	TET	16	244	74
	TMP	34	265	102

Supplementary Table 6.
Percentages of the AMR-associated accessory genes, core genome genes, and overall number of genes, identified by machine learning, that are shared between the Chinese co-inhabiting *E. coli* and *S. enterica* isolates from China (serving as the reference) and the Chinese isolates of not confirmed co-inhabiting *E. coli* and the European isolates of *E. coli* and *S. enterica* (EFFORT + ENGAGE). Hypothetical genes were excluded, each column gives the percentages of genes in common between the sets.

Dataset	Antibiotics	Accessory genes	Core genome genes	Overall number of genes
E. coli (EFFORT)	CHL	0.00%	14.71%	14.71%
	CIP	4.17%	15.71%	15.08%
	NAL	2.70%	13.21%	13.39%
S. enterica (ENGAGE)	CTX	0.00%	1.79%	1.90%
	TET	2.44%	3.66%	3.48%
	TMP	2.44%	3.35%	3.65%

Supplementary Notes

Supplementary Note 1:

Construction of the phylogenetic tree

To construct the phylogenetic tree, the following steps were taken. The serotyping of the *E. coli* isolates revealed 246 distinct serotypes, with varying numbers of isolates per serotype, ranging from 1 to 11. Due to the limited number of isolates within each serotype, we were unable to assess clustering within serotypes. Therefore, we conducted an assessment of isolate clustering based on larger phylogroups to draw more meaningful conclusions. Isolates were primarily from phylogroups A (n=181) or B1(n=221), both associated with commensal *E. coli*, however all other phylogroups were also seen in smaller numbers: C (n=10), D (n=32), E (n=28), F (n=31), G (n=9), Clade I (n=2), Unknown (n=4). Phylogroup prediction was done using in silico Clermont typing[2] based on primer sequences to 15 genes. In contrast, the phylogenetic tree was constructed using the alignment of 3118 genes in the core genome, with only branches with > 95% likelihood kept (ultrafast bootstrap probability). As a result of the application of these two different methodologies, occasionally there were isolates that are predicted to be in one phylogroup by Clermont typing but were overall more similar to isolates within another phylogroup, so they would be placed within a different phylogroup in the tree.

Supplementary Note 2:

Cluster network analysis

To further assess the genomic relatedness of both the *E. coli* and *S. enterica* isolates in our cohort we measured the number of different core genome SNPs, in a pairwise manner across all isolates. Creating a network based on clusters of related isolates with less than 15 SNPs, as done previously[1, 3]. We observed again differences between the two species, with *S. enterica* forming larger clusters compared to *E. coli*, highlighting the greater clonality of the *S. enterica* isolates. For *S. enterica* (**Fig. S14A**), ten large clusters (mean SNPs difference within cluster = 6, range 0-10) were mainly defined by serotype, with poor intermixing of timepoints, whilst the source types were largely intermixed (91%), however an enrichment for ceca samples in Havana isolates is observed, as in the phylogeny. For the *E. coli* (**Fig. S14B**) ninety smaller clusters of highly related isolates (mean SNPs difference within cluster = 4, range 0-15) were present and 71% of these small clusters were composed of more than one sample type. The larger clusters shown in **Fig. S14** are likely due to the larger population structure for *S. enterica* compared to *E. coli.*

As phylogroups can encompass large genomic variations that might be related to geographic distance or time, we analysed the clustering pattern of *E. coli* and *S. enterica* strains using a core genome distance of <100 (SNPs) as done previously[4]. Overall, we found that isolates, of both species, from the same farm were generally more closely related than those of different farms, similarly, isolates from the same province were more closely related than those further apart, **Fig S15**. Analogously, when isolate pairs were compared from different collection dates, we found that strain-level diversity can persist over time and that there was a general trend of increasing SNP distance the further apart the sampling, **Fig. S16**.

Supplementary Note 3:

The challenge of obtaining controls for studying the influence of co-inhabitation

Firstly, we would like to comment on the challenge of obtaining a control group with demonstrated absence of co-inhabitation. To appreciate the challenge, the opposite one, i.e.,

collecting a sample with demonstrated co-inhabitation, must be discussed first. It is difficult to choose a moment of collection, as we need to make sure that both bacterial species are present in the sample at the same time point. The absence of a species in a collected sample may not mean absence of co-inhabitation, but simply be due to the second species having been present earlier (disappeared at the moment of collection, for example due to usage of antibiotics, or simply not being present in sufficient quantities at the time of culturing). There are further challenges related to processing samples with co-inhabiting species, as AST and sequencing data must be generated for all the targeted isolates in parallel. To date, we are not aware of other studies in the literature where the characterisation of co-inhabiting bacterial species in chicken has been attempted, us being the first to do so with our Chinese samples. Under this perspective, it is clear that the demonstration of absence of a species in a sample is an even greater challenge, again because it is not easy to know what happened to the sample ahead of collection, and what would happen to it if the collection were to take place at a later moment in time. A way to strengthen a proof of absence of co-inhabitation would be to collect multiple times at multiple time points, minimising the likelihood of colonisation events that may go undetected. This approach of frequent collection is however difficult to implement, due to the costs and the logistics of a more frequent collection (in our study we were able to collect at only three time points over 42 days).

Creation of control sets and related analyses for co-inhabitation and country of collection

To test for the hypothetic influence on our results (proportion of plasmids and mobile ARGs, shared or not) of factors such as country of collection, as well as of co-inhabitation within the same sample of *S. enterica* and *E. coli*, we created three control sets. The first was formed by considering the Chinese *E. coli* and *S. enterica* isolates collected from bird-related samples where no co-inhabitation was found. Note that the absence of co-inhabitation is not demonstrated, but rather the result of not having found both bacteria in the analysed sample. Then, to obtain further control sets, we searched for *E. coli* and *S. enterica* from poultry in other countries where the usage of antibiotics and/or microbial ecology is different and where the type of interventions (vaccines against *S. enterica*) reduces co-inhabitation of *E. coli* and *S. enterica*. Our search included: (i) extensive literature review in PubMed (using the Boolean search terms "Escherichia coli"[All Fields] AND "poultry" AND "whole genome"[All Fields] and "salmonella"[All Fields] AND "poultry" AND "whole genome"[All Fields]), and (ii) searches on public databases such as BV-BRC, European Nucleotide Archive, NCBI Pathogen Browser for other comparable studies from which whole genomes sequences and phenotypic antibiotic resistance data from chicken faeces could be retrieved. We found two suitable studies with both whole genomes sequences and phenotypic antibiotic resistance data from chicken faeces, taken from countries known to feature a lower likelihood of co-inhabitation. These studies were: the EU project “EFFORT against AMR”[5] and the “ENGAGE” study[6]. The isolates included in both these studies had been collected from countries with strict antimicrobial usage policies, different from those enacted in China.

We collected our first European control group from “EFFORT against AMR”, which provided us with 206 *E. coli* isolates from chicken faeces with AMR phenotype data. These isolates had been obtained from samples taken from chickens in five different European countries (Denmark, Germany, Switzerland, Poland, and Spain) all subjected to the EU Salmonella control measures Regulation (EC) No. 200/2012[7] resulting in strong Salmonella control/vaccination programmes and less than 0.5% Salmonella positive flocks in commercial broiler chickens. We therefore assumed that the isolates retrieved from “EFFORT against AMR” would be much less likely to feature co-habiting *S. enterica* compared to our samples.

The second European control group was collected from the "ENGAGE" study[6] and provided us with a collection of 92 *S. enterica* chicken cecum and faeces isolates from Italy. Given that *E. coli* is a ubiquitous resident/commensal in the chicken gut, we cannot exclude the presence of co-inhabiting *E. coli* in the birds, therefore we labelled this set as "not-necessarily co-inhabiting". Nevertheless, the set was interesting because ecologically distant from both the other European set, and our original Chinese set, in particular as Italy features a much higher proportion of antimicrobial resistance in poultry than the EU average, but lower than China[8, 9, 10].

In the following, we illustrate the analyses done using the new sets. The focus is on identifying changes in identified mobile genetic elements – MGEs (distinct plasmid types and mobile ARGs) found across the different cohorts.

MGEs in Chinese S. enterica isolates: co-inhabiting vs not co-inhabiting

We identified a total of 25 distinct plasmid types in the 70 Chinese co-inhabiting *S. enterica* isolates gathered from chicken faeces and cecal samples. On the contrary, the Chinese *S. enterica* isolates with no confirmed co-inhabitation (n=13), also gathered from chicken faeces and cecal samples, exhibited 14 distinct plasmid types, with all 14 of them being in common with the co-inhabiting set. Of these 14, none were found in a significantly different proportions in either cohort (Proportion test, two-tailed, Bonferroni correction, all adjusted p-values > 0.05).

When comparing the mobile ARGs between the two cohorts, a total of 22 mobile ARGs were identified in the Chinese co-inhabiting isolates from faeces and cecal samples, and 17 were identified in the Chinese non co-inhabiting isolates from faeces and cecal, with all 17 of those of them being in common to both. Of these 17 common mobile ARGs, one was significantly more frequent in the non co-inhabiting cohort (IS6100-*aadA7*) whilst another was significantly more frequent in the co-habiting cohort, ISKpn19-*QnrS1* (Proportion test, two-tailed, Bonferroni correction, all adjusted p-values < 0.05).

MGEs in Chinese E. coli isolates: co-inhabiting vs not co-inhabiting

We identified a total of 45 distinct plasmid types in the 70 Chinese co-inhabiting *E. coli* isolates gathered from chicken faeces and cecal samples. The Chinese *E. coli* isolates with no confirmed co-inhabitation (n=245), gathered chicken faeces and cecal samples, exhibited 52 distinct plasmid types, with 41 of them being in common with the co-inhabiting set. Of these 41, none were found in a significantly different proportions in either cohort (Proportion test, two-tailed, Bonferroni correction, all adjusted p-values > 0.05).

When comparing the mobile ARGs between the two cohorts, a total of 108 mobile ARGs were identified in the Chinese co-inhabiting cohort from faeces and cecal isolates, 155 were identified in the Chinese non co-inhabiting isolates from faeces and cecal, 82 of them being in common to both. Of these 82, none were found in a significantly different proportion in either cohort (Proportion test, two-tailed, Bonferroni correction, all adjusted p-values > 0.05).

MGEs: Chinese S. enterica co-inhabiting isolates vs ENGAGE S. enterica not-necessarily co-inhabiting isolates

When considering the European *S. enterica* isolates (n=92) from Italy (ENGAGE study), 13 distinct plasmid types were found. The Chinese *S. enterica* chicken gut isolates (n=70) from our study (i.e., isolates from samples with confirmed co-inhabitation), exhibited 25 distinct plasmid types, of which 8 in common with the European cohort. Considering the overall number of distinct plasmid types found in the two cohorts, the Chinese cohort carried a significantly greater

number of plasmid types compared to the European isolates (Proportion test across all samples, two-tailed, p value < 0.01). Out of the eight plasmids that overlapped, 2 were significantly more prevalent (Proportion test, two-tailed, Bonferroni correction) in the Chinese isolates compared to the European isolates; namely: Col156 (adj. p-value < 0.0001) and ColVC (adj. p-value < 0.0001). Conversely, four plasmid types were found in significantly higher proportions among the European isolates (IncX1, IncX3, IncX4 and IncFIB(K)), (Proportion test, two-tailed, Bonferroni correction, all adjusted p-values > 0.001). In total, 24 mobile ARG types were found in the ENGAGE cohort, whilst 22 mobile ARGs were found in the Chinese one, none in common between both cohorts. In conclusion, from this comparison the number of plasmid and mobile ARG types is significantly different between the Chinese cohort (*S. enterica* co-inhabiting with *E. coli*) and the ENGAGE cohort (*S. enterica* isolates not necessarily co-inhabiting with *E. coli*).

MGEs: Chinese E. coli co-inhabiting isolates vs EFFORT E. coli not co-inhabiting isolates

We identified a total of 50 distinct plasmid types in the 206 European *E. coli* isolates gathered from the European set. The Chinese *E. coli* gut isolates (n=70) from our study (again taken from samples with confirmed co-inhabitation) exhibited 45 distinct plasmid types, 42 of them being in common with the EFFORT set. Of these 42, six (IncHI2A, IncHI2, pKPC-CAV1321, IncI2(Delta), IncN, IncFII(pHN7A8)) were found in a significantly greater proportion in the Chinese cohort compared to the European cohort (Proportion test, two-tailed, Bonferroni correction, all adjusted p-values < 0.05,Three of the 42 shared plasmid types (IncI1-I(Alpha), ColRNAI, Col(MG828)) were present in a significantly greater proportion in the European cohort compared to the Chinese one.

When comparing the mobile ARGs between the two cohorts, a total of 77 mobile ARGs were identified in the EFFORT cohort and 108 in the Chinese cohort, 22 of them being in common to both. Of the 22 common mobile ARGs, six were significantly more frequent in the Chinese cohort, namely: IS6100-*mphA*, ISEc59-*APH(4)-Ia*, ISEc59-*AAC(3)-IV*, ISKpn19-*QnrS1*, ISVsa3-*floR* (Proportion test, two-tailed, Bonferroni correction, all adjusted p-values < 0.0001). However, ISVsa3-*sul2* was found significantly more frequently in the European cohort (Proportion test, two-tailed, Bonferroni correction, adjusted p-value < 0.001).

Shared MGEs: set of MGEs shared by co-inhabiting Chinese E. coli and Chinese S. enterica vs. set of MGEs shared by EFFORT E. coli and ENGAGE S. enterica

We investigated the possibility of detecting similarities between proportions of distinct plasmid types and mobile ARGs shared by *E. coli* and *S. enterica* isolates, when comparing Chinese and European cohorts. The European cohort consisted of the aggregation of the EFFORT and ENGAGE sets. Larger numbers of shared plasmid types and mobile ARGs were found in the Chinese cohort compared to the European one: 30% vs 26% distinct plasmid types; 16% vs 4% mobile ARGs, though no statistically significant differences were present.

Shared MGEs: set of MGEs shared by co-inhabiting Chinese E. coli and Chinese S. enterica vs. set of MGEs shared by co-inhabiting Chinese E. coli and not-necessarily co-inhabiting ENGAGE S. enterica

Both plasmid types and mobile ARGs were found significantly more shared when analysing the Chinese isolates (*S. enterica* + *E. coli* isolates), compared to the combined set formed by ENGAGE *S. enterica* isolates and Chinese *E. coli* isolates (two-tailed chi-squared test with Yates and Bonferroni correction, adjusted p-value = 0.05).

Overall, our results show two things:

a) There is a positive correlation between co-inhabitation of *E. coli* and *S. enterica* and the proportions of mobile genetic elements (plasmids and mobile ARGs) observed in the isolates (shared and not shared between the two bacterial species), as indicated in particular by the comparison of the Chinese cohorts with confirmed and not confirmed co-inhabitation, and by the comparison of Chinese *E. coli* isolates (with confirmed co-inhabitation) with the EFFORT *E. coli* isolates (with likely absence of co-inhabitation, due to the very low Salmonella prevalence in the five EU countries considered, due to the EU Salmonella control measures Regulation (EC) No. 200/2012 (European Food Safety Authority, 2018) resulting in strong Salmonella control/vaccination programmes and less than 0.5% Salmonella positive flocks in commercial broiler chickens).
b) There is a higher occurrence of mobile genetic elements within the individual species (*S. enterica* and *E. coli*) in our Chinese cohort compared to the European cohorts, Analogously, we observe a greater prevalence of shared MGEs between the two species in the Chinese cohort, compared to the European ones. These observations indicate a likely influence of the country of collection.

Supplementary Note 4:

Influence of datasets (country of collection, co-inhabitation) on ML results

To investigate whether the results (identified genetic elements associated with AMR) predicted by the machine learning pipeline trained with the Chinese *E. coli* and *S. enterica* cohorts (with confirmed co-inhabitation of the bacterial species) would change if the same pipeline was trained with different cohorts (e.g., from another country, and/or with no co-inhabitation) we performed a further investigation by training the machine learning pipeline multiple times. In the "test" runs, using Chinese *E. coli* and *S. enterica* isolates (with confirmed co-inhabitation) for training; in the "control" runs, using the Chinese not co-inhabiting chicken isolates and the European datasets retrieved from the EFFORT and ENGAGE projects (previously described in **Supplementary Note 3**). Then, we compared the results to observe the degree of overlap of the predictions (identified genetic elements associated to AMR).

Methods and datasets

We first selected the set of *E. coli* and *S. enterica* isolates that had been confirmed to be co-inhabitants in our Chinese cohort. This set consisted of instances in which positive cultures verified the presence of both *E. coli* and *S. enterica* species in chicken, with a total of 98 such cases. The 98 isolates included: 47 chicken faeces, 23 chicken caecal droppings, 16 chicken carcass, 12 chicken feather. Since the number of confirmed co-inhabiting isolates in chicken faeces alone was limited to 47, insufficient for training the machine learning model, and by including chicken caecal droppings we increase to 70 (still insufficient), we opted to perform the training using all confirmed co-inhabiting isolates found in chicken sources (including faeces, ceca, carcasses, and feathers). Then, we selected the not co-inhabiting isolates from the same Chinese cohort of chicken isolates. Next, we selected the data from the European EFFORT and ENGAGE sets to perform an analogous training of the machine learning models, in the control experiments.

The same machine learning pipeline (see **Methods** in the manuscript) was used in both the test experiments (Chinese *E. coli* and Chinese *S. enterica* data) and control experiments (not co-inhabiting Chinese and European data).

Machine learning results for the Chinese co-inhabiting sets

When training the machine learning models on the Chinese data, the only occasional modification to the pipeline was the p-value of the chi-square test, set to 0.05 for *E. coli* due to the small number of isolates. The following features were encoded as inputs to the machine learning models: presence/absence of accessory genes, core genome SNPs and intergenic region SNPs (therefore covering both mutations and genes). When training the machine learning models for the test experiments (Chinese data), for some of the 28 antimicrobials, because of the limited number of isolates, the distribution of resistance/susceptibility profiles was skewed, with most isolates being either susceptible or resistant. Consequently, these data could not be used for constructing predictive models. Given the above limitations we were able to run the machine learning pipeline on 3 antibiotics (chloramphenicol - CHL, ciprofloxacin - CIP, nalidixic acid – NAL) for the co-inhabiting *E. coli* dataset and on 5 antibiotics (cefotaxime – CTX, gentamycin – GEN, minocycline – MIN, tetracycline – TET and trimethoprim / sulfamethoxazole – SXT) for the co-inhabiting S. *enterica* dataset. **Figs. S17 and S18** show the performance metrics and the number of features for the co-inhabiting *E. coli* and *S. enterica* Chinese datasets. The performance metrics for all classifiers are in **Supplementary Data 6**, while the selected features for each antibiotic model are in **Supplementary Data 12**.

The application of the remaining part of the pipeline, led to the results illustrated in **Supplementary Table 2** for the Chinese datasets.

Machine learning results for the Chinese not co-inhabiting sets

When training the machine learning models on the Chinese not co-inhabiting sets, no modification was done to the original proposed pipeline. There were 337 not co-inhabiting *E. coli* isolates from chicken sources, a sufficient number to train the machine learning pipeline to create predictor models for *E. coli*. However, there were only 17 not co-inhabiting *S. enterica* isolates from chicken sources, which was not sufficient to train the machine learning models. Therefore, the analyses focused solely on training the machine learning models for *E. coli*. The same features used in the test experiments (with co-inhabitant isolates) were encoded as inputs, i.e., presence/absence of accessory genes, core genome SNPs and intergenic region SNPs. The predictive models for some of the antibiotics could not be trained, because of excessive disproportion of resistance or susceptibility phenotypes for those antibiotics. We were able to complete the training for 15 antibiotics (amikacin – AMI, aztreonam – AZM, ceftazidime - CAZ, ceftazidime/clavulanic acid – CAZ-C, cefoxitin – CFX, chloramphenicol - CHL, cefotaxime – CTX, cefepime – FEP, gentamycin – GEN, kanamycin – KAN, minocycline – MIN, nalidixic acid – NAL, streptomycin – STR, sulphafurazole – SUL and trimethoprim/sulfamethoxazole – SXT). The performance metrics and the number of features selected as relevant by the machine learning predictors of AMR to specific antibiotics in *E. coli*, are summarised in **Fig. S19**. These models ran using the not co-inhabiting *E. coli* isolates. The performance metrics for all classifiers are in **Supplementary Data 6**, while the selected features for each antibiotic model are in **Supplementary Data 13**.

The application of the pipeline, led to the results illustrated in **Supplementary Table 3** for the Chinese not co-inhabiting datasets.

Comparison between Chinese not co-inhabiting and Chinese co-inhabiting machine learning results

The comparison between not co-inhabiting and co-inhabiting results is summarised in **Supplementary Table 4**, where the degree of overlap of identified genetic elements is illustrated as percentages. The overlaps (i.e., percentages of genes identified by machine learning in both datasets) were estimated by mapping the findings back to the pangenome. Note that in the table, the co-inhabiting sample (test) is taken as the reference (as opposed to selecting the control, i.e. the not co-inhabiting sample). This is to allow comparison of overlaps with the following tests. Despite the overlaps in **Supplementary Table 4** being in some cases relatively large, the differences in prediction generated by the machine learning models provide an additional hint at co-inhabitation having a likely effect on the machine learning prediction.

An average of 39.37% of genes were found in common across antibiotics. This includes both accessory and core genes. In more detail, 723 genes were found in common, representing accessory genes and core genes related to SNPs across all antibiotic modes. Interestingly, only five of these genes are known antibiotic resistance genes: *kpdE* (aminoglycoside), *emrB* (fluoroquinolone), *mdtA* (multi drug resistance), *cpxA* (multi drug resistance), *acrB* (multi drug resistance).

Machine learning results for the European not-necessarily co-inhabiting sets

As anticipated earlier, a second set of experiments was conducted using machine learning, to incorporate in the analysis the investigation of a possible influence of country of collection. The aforementioned EFFORT and ENGAGE datasets were used to train machine learning models in order to obtain further control results. It should be reiterated that the EFFORT *E. coli* set is labelled as "not co-inhabiting" due to the less likely presence of *S. enterica* consequent to bird treatment; whilst the ENGAGE *S. enterica* set is labelled as not-necessarily co-inhabiting because the presence of *E. coli* is unknown.

For *E. coli* (EFFORT dataset) we had 206 isolates with AMR phenotype data, of which 7 antibiotics (ampicillin - AMP, chloramphenicol - CHL, ciprofloxacin - CIP, nalidixic acid – NAL, sulfisoxazole – SUL, tetracycline – TET and trimethoprim – TMP) had enough isolates in each class for the machine learning models to be trained. For *S. enterica* (ENGAGE dataset) 92 isolates were available with AMR phenotype data, of which 5 antibiotics (ampicillin – AMP, chloramphenicol – CHL, cefotaxime – CTX, tetracycline – TET, trimethoprim – TMP) had enough isolates in each class for the machine learning models to be trained. **Figs. S20 and S21** and **Supplementary Table 5**, report the performance metrics and the features identified by the machine learning pipeline for the control experiments. The performance metrics for all classifiers are in **Supplementary Data 5**, while the selected features for each antibiotic model are in **Supplementary Data 14**.

Comparison between European not-necessarily co-inhabiting and the Chinese co-inhabiting machine learning results

In **Supplementary Table 6**, the degrees of overlap between the results on the European sets and the original results obtained in the Chinese co-inhabitant set are shown. As the **Supplementary Table 6** shows, the degrees of overlap are very low, indicating a likely influence of country of collection, although the result may also be influenced by a likely lesser co-inhabitation featured in the European sets. Also, note that the results (overlaps) in **Supplementary Table 4** and **Supplementary Table 6** are comparable, as they have been computed using the same reference (results found using the co-inhabitant Chinese set).

A lower overlap (14.19%) was observed when comparing EFFORT not co-inhabiting *E. coli* with Chinese co-inhabiting *E. coli*, compared to Chinese not co-inhabiting *E. coli* vs Chinese co-inhabiting *E. coli*. (39.37%). In the comparison involving the EFFORT *E. coli*, 246 genes were found in common, representing accessory genes and core genome genes related to SNPs across all antibiotic models. Interestingly, only four of these genes are known antibiotic resistance genes: *kpdE* (aminoglycoside), *emrB* (fluoroquinolone), *mdtA* (multi drug resistance), *eptA* (peptide).

Lower rates of overlap were observed when comparing the results for the ENGAGE not-necessarily co-inhabiting *S. enterica* isolates with the Chinese co-inhabiting *S. enterica* isolates. On average, only 3.01% of genes were shared across three antimicrobial models, with the following antibiotic-specific percentages: TMP = 3.65%, CTX = 1.90%, and TET = 3.48%. In more detail, 55 genes were found in common, representing accessory genes and core genome genes related to SNPs across all antibiotic models. There was only one known antibiotic resistance gene (*oprM*), which is actually associated with multi-drug resistance.

The scenario presented by **Supplementary Table 4** and **Supplementary Table 6** indicates that both country of collection and presence/absence of co-inhabitation have an influence in the genetic elements discovered by machine learning. Note that these results are consistent with what illustrated in the previous response for the more conventional analysis of MGEs (i.e., country of collection appears to be more influential than presence/absence of co-inhabitation). The results are particularly consistent with the possible differences existing in the microbial ecosystems between Chinese and European anthropogenic settings, driven by variations in antibiotic usage, intervention strategies (such as vaccines against *Salmonella*). There is a possibility also that country-related differences may influence the rate of co-inhabitation, possibly shedding some light on the currently unknown co-inhabitation situation in the ENGAGE *S. enterica* dataset. Nevertheless, we acknowledge that our analysis may be influenced by further confounding effects. To better explore and separate potential confounding factors a long-term evolutionary case-control study would be required.

Supplementary Note 5:

Preliminary experimental validation of the machine learning results

To obtain some experimental validation on the results obtained by running the ML method, we selected three genes from the top-ranked 10% identified by the pipeline, using the following criteria: a) present in the *E. coli* and *S. enterica* co-inhabiting set; b) harbouring non-synonymous SNPs; c) significantly impacting reaction flux when knocked out, as highlighted by the genome-scale metabolic model; d) availability of the knockout strain. This resulted in the three candidate genes, top-ranked by ML for the antibiotics ampicillin and doxycycline, namely: *hisA* (obtained by mapping the SNP: P109Q back onto the genome), *argI* (SNP: A153T) and *fhuB* (SNP: N47D). Interestingly, neither of these three genes is currently present in any AMR databases.

HisA is a $(\beta\alpha)_8$ barrel enzyme that catalyses the Amadori rearrangement of *N′*-[(5′-phosphoribosyl)formimino]-5-aminoimidazole-4-carboxamide ribonucleotide (ProFAR) to *N′*-((5′-phosphoribulosyl) formimino)-5-aminoimidazole-4-carboxamide-ribonucleotide (PRFAR) in the histidine biosynthesis pathway. The amino acid position 109, identified by machine learning as associated to the doxycline resistance phenotype, is found in the histidine biosynthesis domain (1-234). The gene *argI* encodes for catalysing ornithine to produce a substrate L-citrulline for argininosuccinate synthetase that is part of the arginine biosynthesis (ornithine to produce a substrate L-citrulline)[11] . The gene *fhuB* is part of the ferrichrome iron transporter Fhu operon,

containing genes *fhuA*, *fhuB*, *fhuC*, and *fhuF*. The protein FhuB forms the transmembrane complex comprised of ten transmembrane helixes creating a pore for transmembrane transport.

For the experimental validation of these three genes, we used the publicly available Keio gene *E. coli* knockout collection[12]. Bacterial growth was determined by using an automated luminometer-spectrometer (TECAN). Overnight cultures of *E. coli* strains were diluted 1:1000 in fresh lysogeny broth (LB), and 0.2 ml cultures were grown at 35°C in 96-well microtitre plates. Turbidity was measured as optical density at 600 nanometres (OD600). Antimicrobial susceptibility to ampicillin and doxycycline was determined by broth microdilution and interpreted according to the Clinical & Laboratory Standards Institute interpretive criteria (CLSI 2009). *E. coli* ATCC 25922 was used as a control for the antimicrobial susceptibility testing.

Both *argI* and *fhuB* mutants exhibited increased antimicrobial susceptibility compared to the parental wild type, as shown by a reduced yield after 20 hours of incubation in LB in the presence of ampicillin at a concentration of 3.55 µg/ml with a confidence level of 99.9% (**Fig. S22**). Similarly, the *hisA* mutant failed to reach the same optical densities as the wild type in the presence of doxycycline over a range of concentrations between 0.19 and 1.77 µg/ml with a confidence level of 99% (**Fig. S22**). While this analysis is limited to only knocking out genes rather than introducing mutations, these results give a preliminary experimental support for possible the involvement of these genetic elements as AMR-determinants, as predicted by machine learning. It is important to recognise that none of the experimentally observed changes in growth were sufficient to cause a change to the resistance/susceptibility classification of the *E. coli* strain. It is likely that individual mutations alone are not strong enough influencers of resistance, but may require the co-presence and co-occurrence of further AMR-associated genetic determinants[13, 14].

Supplementary Note 6:

Proposed machine learning pipeline compared to existing GWAS approaches

Recent works published by us and others have demonstrated that culture based approaches with whole genome sequencing of individual pathogens, antibiotic susceptibility testing and ML techniques are effective predictors of genomic characteristics linked to AMR for both *E. coli* isolates[3, 15, 16, 17] and other bacteria [18, 19, 20, 21, 22, 23]. Therefore, this work developed a methodology based on ML to accurately associate multiple genomic features (accessory genes, core genome SNPs and intergenic region SNPs) with *E. coli* and *Salmonella* antibiotic resistances. Compared to conventional GWAS methods only processing a single genetic element type at each run (most frequently SNPs), our method simultaneously considers SNPs and accessory genes, allowing to capture complex interactions in relation to both mutation and horizontal gene transfer. The use of machine learning allows for better handling problems with large numbers of features compared to the number of observations, and can better cope with multi-collinearity of in the feature set, while also providing tools for ranking and filtering the identified features based on influence on the phenotype.

Confounding factors possibly influencing the illustrated results

It is important to discuss potential confounding factors, unaccounted for in our analysis, which may have influenced our results. Amongst those more likely to be influential, we can list:

- Factors related to the place of collection, for example: a) differences in rearing practices between the covered Chinese regions and farms (including feed, antibiotic treatment, use of net housing vs cage housing, etc.), with the added complexity that some of these practices may be poorly documented; b) differences in environmental variables, such as

temperature and humidity). For example, in recent works[24, 25] we have demonstrated that there is a correlation between AMR in *E. coli* in the chicken gut, and environmental temperature and humidity in the barn; c) other region-related differences, possibly even more difficult to account for (for example differences in local microbial ecologies across regions).

- Factors related to health and welfare of the birds. Note that our samples were pooled, thus averaging the conditions of the birds in the net or cage of collection. However, there likely is still bias due to this factor, as our sample size (977 samples over 10 farms) is very little considering that a typical Chinese barn may host between 12k and 33k birds, a farm usually has multiple barns, the collection covered 10 farms, and there are 6-7 weeks of bird life + slaughtering, which in this research were sampled in only three time points.
- Issues in sample collection and analysis. For example, when culturing it is very difficult to demonstrate absence of co-inhabitation of the two bacterial species *E. coli* and *S. enterica*, as such conclusion was inferred when one of the two species was not found in the sample (when in reality the species might have been present earlier in time, or in undetectable quantities – see elsewhere in this discussion). Several other confounding factors may be involved in the analysis.

There will clearly be many other factors that have not been explicitly listed above. Whilst the effects of the country of origin have been touched upon, with quantitative experiments (see Results and Supplementary Material, and a similar analysis was run on various degrees of likelihood of absence of co-inhabitation, also in the supplementary material), several others of the factors listed above, as well as many unlisted others, remain unaddressed. Clearly the main limitation is in the size/coverage our sample, which was sufficient to support the presented conclusions, but insufficient in terms of size and diversity, to guarantee sufficient coverage of all the potential conditions that should be investigated in order to isolate the many factors that are surely involved in the study of genetic convergence of AMR.

Supplementary References

1. Ludden C*, et al.* One health genomic surveillance of *Escherichia coli* demonstrates distinct lineages and mobile genetic elements in isolates from humans versus livestock. *mBio* **10**, e02693-02618 (2019).

2. Beghain J, Bridier-Nahmias A, Le Nagard H, Denamur E, Clermont O. ClermonTyping: an easy-to-use and accurate in silico method for Escherichia genus strain phylotyping. *Microbial genomics* **4**, e000192 (2018).

3. Peng Z*, et al.* Whole-genome sequencing and gene sharing network analysis powered by machine learning identifies antibiotic resistance sharing between animals, humans and environment in livestock farming. *PLoS Comput Biol* **18**, e1010018 (2022).

4. Shaw LP, *et al.* Niche and local geography shape the pangenome of wastewater- and livestock-associated Enterobacteriaceae. *Sci Adv* **7**, (2021).

5. Leekitcharoenphon P, *et al.* Genomic evolution of antimicrobial resistance in Escherichia coli. *Sci Rep* **11**, 15108 (2021).

6. Alba P, *et al.* Molecular epidemiology of Salmonella Infantis in Europe: insights into the success of the bacterial host and its parasitic pESI-like megaplasmid. *Microb Genom* **6**, (2020).

7. Authority EFS, Prevention ECfD, Control. The European Union summary report on trends and sources of zoonoses, zoonotic agents and food-borne outbreaks in 2017. *EFSA Journal* **16**, e05500 (2018).

8. Vieira AR, *et al.* Association between antimicrobial resistance in Escherichia coli isolates from food animals and blood stream isolates from humans in Europe: an ecological study. *Foodborne Pathog Dis* **8**, 1295-1301 (2011).

9. Allel K, *et al.* Global antimicrobial-resistance drivers: an ecological country-level study at the human-animal interface. *Lancet Planet Health* **7**, e291-e303 (2023).

10. Authority EFS, Prevention ECfD, Control. The European Union Summary Report on Antimicrobial Resistance in zoonotic and indicator bacteria from humans, animals and food in 2020/2021. *EFSA Journal* **21**, e07867 (2023).

11. Thongbhubate K, Irie K, Sakai Y, Itoh A, Suzuki H. Improvement of putrescine production through the arginine decarboxylase pathway in Escherichia coli K-12. *AMB Express* **11**, 168 (2021).

12. Baba T, *et al.* Construction of Escherichia coli K-12 in-frame, single-gene knockout mutants: the Keio collection. *Mol Syst Biol* **2**, 2006.0008 (2006).

13. Johnson JR, Johnston B, Clabots C, Kuskowski MA, Castanheira M. Escherichia coli sequence type ST131 as the major cause of serious multidrug-resistant E. coli infections in the United States. *Clin Infect Dis* **51**, 286-294 (2010).

14. Mobegi FM, Cremers AJ, de Jonge MI, Bentley SD, van Hijum SA, Zomer A. Deciphering the distance to antibiotic resistance for the pneumococcus using genome sequencing data. *Sci Rep* **7**, 42808 (2017).

15. Her H-L, Wu Y-W. A pan-genome-based machine learning approach for predicting antimicrobial resistance activities of the Escherichia coli strains. *Bioinformatics* **34**, i89-i95 (2018).

16. Hyun JC, Kavvas ES, Monk JM, Palsson BO. Machine learning with random subspace ensembles identifies antimicrobial resistance determinants from pan-genomes of three pathogens. *PLoS Comput Biol* **16**, e1007608 (2020).

17. Pearcy N, *et al.* Genome-scale metabolic models and machine Learning reveal genetic determinants of antibiotic resistance in *Escherichia coli* and unravel the underlying metabolic adaptation mechanisms. *mSystems* **6**, e00913-00920 (2021).

18. Kavvas ES, *et al.* Machine learning and structural analysis of *Mycobacterium tuberculosis* pan-genome identifies genetic signatures of antibiotic resistance. *Nat Commun* **9**, 4306 (2018).

19. Kavvas ES, Yang L, Monk JM, Heckmann D, Palsson BO. A biochemically-interpretable machine learning classifier for microbial GWAS. *Nat Commun* **11**, 2580 (2020).

20. ValizadehAslani T, Zhao Z, Sokhansanj BA, Rosen GL. Amino Acid k-mer Feature Extraction for Quantitative Antimicrobial Resistance (AMR) Prediction by Machine Learning and Model Interpretation for Biological Insights. *Biology* **9**, 365 (2020).

21. Wang W, *et al.* Whole-genome sequencing and machine learning analysis of *Staphylococcus aureus* from multiple heterogeneous sources in China reveals common genetic traits of antimicrobial resistance. *mSystems* **6**, e01185-01120 (2021).

22. Wang W, *et al.* Novel SCCmec type XV (7A) and two pseudo-SCCmec variants in foodborne MRSA in China. *J Antimicrob Chemother*, (2022).

23. Liu Z, *et al.* Evaluation of Machine Learning Models for Predicting Antimicrobial Resistance of Actinobacillus pleuropneumoniae From Whole Genome Sequences. *Front Microbiol* **11**, 48 (2020).

24. Maciel-Guerra A, *et al.* Dissecting microbial communities and resistomes for interconnected humans, soil, and livestock. *The ISME Journal*, (2022).

25. Baker M, *et al.* Machine learning and metagenomics reveal shared antimicrobial resistance profiles across multiple chicken farms and abattoirs in China. *Nature Food* **4**, 707-720 (2023).
