# Supplementary material for: Convergence of resistance and evolutionary responses in Escherichia coli and Salmonella enterica co-inhabiting chicken farms in China
Source: Nat Commun. 2024 Jan 5;15:206. doi: 10.1038/s41467-023-44272-1 (PMC10770378; doi:10.1038/s41467-023-44272-1)
Supplement: Supplementary file 3 — Description of Additional Supplementary Files [file 41467_2023_44272_MOESM3_ESM.pdf]

Description of Additional Supplementary Files

Supplementary Data 1.

Characteristics of the *E. coli* and *S. enterica* isolates, including collection details, typing data, assembly statistics, genomic features, and results of antibiotic susceptibility testing and CARD ARGs. The annotation of resistance determinants may change if using a different database. For example, the gene aac(6'')-Iaa is annotated as not conferring resistance in S. enterica when using the AMRFinderPlus database.

Supplementary Data 2.

Alignment lengths and Bayesian parameter outputs including 95% HSP for clock/ UCLD rate, root height and age, for *S. enterica* serotypes Kentucky, Kedougou, Indiana, Havana and Enteritidis and *E. coli* phylogroups A, E and F.

Supplementary Data 3.

Presence-absence of plasmids, separated by replicon type, on each of the 518 *E. coli* and 143 *S. enterica* isolates by comparison to the PlasmidFinder database with 80% identity and 70% coverage thresholds.

Supplementary Data 4.

List of the shared mobile ARGs within the 113 isolate pairs of *E. coli* and *S. enterica* isolates cultured from the same sample. Mobile ARGs are defined as the presence of and MGE within 5kb upstream or downstream of the known ARG. Isolate ID, mobile ARG, farm and source type are given.

Supplementary Data 5.

Pairwise Jaccard coefficients for the resistance profile of each antibiotic compared to the presence of each known AMR gene found in CARD. The source data for the calculations is located in Supplementary Data 1.

Supplementary Data 6.

Machine learning performance for each of the 7 classification methods used for both *E. coli* isolates and *S. enterica* isolates correlating the genetic features (SNPs and accessory genes) to the resistance/susceptibility against a panel of antimicrobials. The performance metrics were accuracy (TP+TN/(P+N)), sensitivity (true positive rate: TP/P), specificity (true negative

rate: TN/N), AUC and precision. The scores for each performance metric were computed from 30 simulations using nested cross-validation. The mean ± standard deviation of these 30 iterations was then used as the result statistics for the performance.

Supplementary Data 7.

Feature importance of genes selected by the ML pipeline as correlated to the resistance-susceptibility of either *E. coli* or *S. enterica* to a panel of 26 antibiotics. The genes listed were either accessory genes or core genes containing a SNP correlated to either resistance or susceptibility. Where the gene was identified as a known AMR gene present in public databases (by comparison using BLAST) the accession is given. Feature importance is given as average importance across models. For each model, the feature importance was calculated based on the Gini Importance of an ExtraTree Classifier with 50 estimators.

Supplementary Data 8.

Core SNP features selected by the ML pipeline as correlated to the resistance-susceptibility of either *E. coli* or *S. enterica* to a panel of 26 antibiotics. For each feature the following information is given: the machine learning Chi squared test (two-tailed) p value (*E. coli* only) and the Gini Importance coefficient; the name of the gene where the SNP is located; a gene description; the amino acid position of the SNP; whether the SNP is synonymous, non-synonymous or nonsense; whether the gene was found to be significant in the GSM model; the StringDB protein annotation; the StringDB gene ontology function; metabolic pathways associated with the gene according with Biocyc.

Supplementary Data 9.

KEGG ontology of the 88 genes found in the top 10% of most important features in both the *E. coli* and *S. enterica* models.

Supplementary Data 10.

Genes used as input to the genome scale models, derived from the top 10% of features in each of the machine learning models.

Supplementary Data 11.

List of genome accessions of European and Chinese public data used in study for comparison with data collected as part of the study.

Supplementary Data 12.

Features selected by the ML pipeline as correlated to the resistance-susceptibility of either *E. coli* to a panel of 3 antibiotics or *S. enterica* to a panel of 5 antibiotics using the Chinese co-inhabiting chicken isolates. The features are either accessory genes, core genome SNPs or intergenic region SNPs. The number "1" indicates that the feature was found in the respective antibiotic model.

Supplementary Data 13.

Features selected by the ML pipeline as correlated to the resistance-susceptibility of *E. coli* to a panel of 15 antibiotics using the Chinese not co-inhabiting chicken isolates. The features are either accessory genes, core genome SNPs or intergenic region SNPs. The number "1" indicates that the feature was found in the respective antibiotic model.

Supplementary Data 14.

Features selected by the ML pipeline as correlated to the resistance-susceptibility of either *E. coli* to a panel of 7 antibiotics or *S. enterica* to a panel of 5 antibiotics using the European not-necessarily co-inhabiting chicken isolates. The features are either accessory genes, core genome SNPs or intergenic region SNPs. The number "1" indicates that the feature was found in the respective antibiotic model.
