# Supplementary material for: Convergence of resistance and evolutionary responses in Escherichia coli and Salmonella enterica co-inhabiting chicken farms in China
Source: Nat Commun. 2024 Jan 5;15:206. doi: 10.1038/s41467-023-44272-1 (PMC10770378; doi:10.1038/s41467-023-44272-1)
Supplement: Supplementary file 18 — Reporting Summary [file 41467_2023_44272_MOESM18_ESM.pdf]

nature portfolio

Corresponding author(s): Tania Dottorini

Last updated by author(s): Nov 27, 2023

Reporting Summary

Nature Portfolio wishes to improve the reproducibility of the work that we publish. This form provides structure for consistency and transparency in reporting. For further information on Nature Portfolio policies, see our Editorial Policies and the Editorial Policy Checklist.

Statistics

For all statistical analyses, confirm that the following items are present in the figure legend, table legend, main text, or Methods section.

n/a	Confirmed	
☐	☒	The exact sample size (*n*) for each experimental group/condition, given as a discrete number and unit of measurement
☐	☒	A statement on whether measurements were taken from distinct samples or whether the same sample was measured repeatedly
☐	☒	The statistical test(s) used AND whether they are one- or two-sided<br>*Only common tests should be described solely by name; describe more complex techniques in the Methods section.*
☒	☐	A description of all covariates tested
☐	☒	A description of any assumptions or corrections, such as tests of normality and adjustment for multiple comparisons
☐	☒	A full description of the statistical parameters including central tendency (e.g. means) or other basic estimates (e.g. regression coefficient) AND variation (e.g. standard deviation) or associated estimates of uncertainty (e.g. confidence intervals)
☐	☒	For null hypothesis testing, the test statistic (e.g. F, t, r) with confidence intervals, effect sizes, degrees of freedom and P value noted<br>*Give P values as exact values whenever suitable.*
☐	☒	For Bayesian analysis, information on the choice of priors and Markov chain Monte Carlo settings
☒	☐	For hierarchical and complex designs, identification of the appropriate level for tests and full reporting of outcomes
☒	☐	Estimates of effect sizes (e.g. Cohen's *d*, Pearson's *r*), indicating how they were calculated

Our web collection on statistics for biologists contains articles on many of the points above.

Software and code

Policy information about availability of computer code

Data collection: No software was used for data collection

Data analysis: The code is available on Github: https://github.com/tan0101/Commercial_WGS2023 under DOI 10.5281/zenodo.10210870

This link has also been added in the “Data and materials availability” section of the manuscript

For the machine learning and bioinformatics data analysis, the following open-source software were used:
Readfq (V10, https://github.com/cjfields/readfq)
SPAdes v3.13
QUAST v4.5
checkM
Prokka v1.14.5
Abricate v1.0.1
MLST v2.19.0
ezclermont 0.6.3
R v3.6.2
BLAST 2.10.1+
LIAN Ver. 3.7
Roary v3.13
Piggy v1.5
IQTree v2.1.4-beta

Matplotlib v3.6.2
Scikit-learn version 1.2.1
Cameo
NetworkX
Python v3.7
iTOLv5
BEAST v 1.10.4
LogCombiner v1.10.4
TreeAnnotator v1.10.4
Tracer v1.7.1
snippy v.4.4.5
EasyFig v2.2.5

For manuscripts utilizing custom algorithms or software that are central to the research but not yet described in published literature, software must be made available to editors and reviewers. We strongly encourage code deposition in a community repository (e.g. GitHub). See the Nature Portfolio guidelines for submitting code & software for further information.

Data

Policy information about availability of data

All manuscripts must include a data availability statement. This statement should provide the following information, where applicable:

- Accession codes, unique identifiers, or web links for publicly available datasets
- A description of any restrictions on data availability
- For clinical datasets or third party data, please ensure that the statement adheres to our policy

Source data are provided with this paper.
The sequencing data supporting the conclusions of this article are available in the NCBI database under BioProject accession numbers:
PRJNA675772 (Shandong 1_1 and 1_2) available at: https://www.ncbi.nlm.nih.gov/bioproject/PRJNA675772

PRJNA841811 (E. coli WGS from other farms) available at:
https://www.ncbi.nlm.nih.gov/bioproject/PRJNA841811

PRJNA841813 (S. enterica from all farms):
https://www.ncbi.nlm.nih.gov/bioproject/PRJNA841813

Public datasets were obtained from the European Nucleotide Archive with accession numbers listed in Supplementary Data 11.

Research involving human participants, their data, or biological material

Policy information about studies with human participants or human data. See also policy information about sex, gender (identity/presentation), and sexual orientation and race, ethnicity and racism.

Reporting on sex and gender

Use the terms sex (biological attribute) and gender (shaped by social and cultural circumstances) carefully in order to avoid confusing both terms. Indicate if findings apply to only one sex or gender; describe whether sex and gender were considered in study design; whether sex and/or gender was determined based on self-reporting or assigned and methods used.
Provide in the source data disaggregated sex and gender data, where this information has been collected, and if consent has been obtained for sharing of individual-level data; provide overall numbers in this Reporting Summary. Please state if this information has not been collected.
Report sex- and gender-based analyses where performed, justify reasons for lack of sex- and gender-based analysis.

Reporting on race, ethnicity, or other socially relevant groupings

Please specify the socially constructed or socially relevant categorization variable(s) used in your manuscript and explain why they were used. Please note that such variables should not be used as proxies for other socially constructed/relevant variables (for example, race or ethnicity should not be used as a proxy for socioeconomic status).
Provide clear definitions of the relevant terms used, how they were provided (by the participants/respondents, the researchers, or third parties), and the method(s) used to classify people into the different categories (e.g. self-report, census or administrative data, social media data, etc.)
Please provide details about how you controlled for confounding variables in your analyses.

Population characteristics

Describe the covariate-relevant population characteristics of the human research participants (e.g. age, genotypic information, past and current diagnosis and treatment categories). If you filled out the behavioural & social sciences study design questions and have nothing to add here, write "See above."

Recruitment

Describe how participants were recruited. Outline any potential self-selection bias or other biases that may be present and how these are likely to impact results.

Ethics oversight

Identify the organization(s) that approved the study protocol.

Note that full information on the approval of the study protocol must also be provided in the manuscript.

Field-specific reporting

Please select the one below that is the best fit for your research. If you are not sure, read the appropriate sections before making your selection.

☒ Life sciences ☐ Behavioural & social sciences ☐ Ecological, evolutionary & environmental sciences

For a reference copy of the document with all sections, see nature.com/documents/nr-reporting-summary-flat.pdf

Life sciences study design

All studies must disclose on these points even when the disclosure is negative.

Sample size	For the analysis of how E. coli and S. enterica are differentiated (in relation to phylogenetic analysis, SNPs distance and AST) across farm and abattoir sources, time-points and geographical locations we did not perform sample size calculation as this was an observational/exploratory study.<br>For the ML analysis, we performed a sample size calculation as detailed in the following. For the results demonstrating the proposed approach to the AMR prediction (based on ML analysis of resistant phenotypes), sample size is based on achieving desired power in the predictor. For binary classifiers, power is the sensitivity (true positive rate, defined as 1-beta, where beta is the false negative rate, i.e. type II error (Banerjee, A. et al. 2009, Industrial psychiatry journal). Note that the type II error is particularly relevant for resistance, as it implies a resistant phenotype escaping detection (Mahfouz, N. et al. 2020, Journal of Antimicrobial Chemotherapy). Using 518 E. coli samples, we achieved an average power of 88% for the 21 antibiotic models studied and using 143 S. enterica samples, we achieved an average power of 94% for the 13 antibiotic models studied. We also wanted to identify the minimum number of samples required to achieve at least 80% sensitivity (power). Because for classifiers based on ML (e.g. SVMs, decision trees, random forest, adaboost, neural networks), sample size calculation to achieve power is not directly possible using conventional analytical methods (Li. J. et al. 2020, Patterns), we applied a bespoke iterative method (wrapper backward selection - WBS, Figueroa, R et al. 2012, BMC Medical Informatics and Decision Making) as done in our previous paper (Maciel-Guerra, A. et al. 2022, The ISME Journal). The method estimates how power decreases with smaller sample sizes. In our case, WBS estimated the need of 419 samples on average for E. coli and 106 samples on average for S. enterica, to achieve 80% power, which is less to what we used.
Data exclusions	No data were excluded.
Replication	At least two/three biological replicates per sample were taken. For the experimental validation (knock-out and wild-type E .coli K12) of the AMR-related genetic elements we used three biological replicates. All replicated were successful.
Randomization	Biological samples were collected randomly without knowing the AMR phenotypes.<br>For the ML classification the samples were randomly assigned to training and testing groups using a nested cross validation procedure (30 iterations per classifier).
Blinding	Biological samples were collected randomly without knowing the AMR phenotypes.

Reporting for specific materials, systems and methods

We require information from authors about some types of materials, experimental systems and methods used in many studies. Here, indicate whether each material, system or method listed is relevant to your study. If you are not sure if a list item applies to your research, read the appropriate section before selecting a response.

Materials & experimental systems

n/a	Involved in the study	
☒	☐	Antibodies
☒	☐	Eukaryotic cell lines
☒	☐	Palaeontology and archaeology
☒	☐	Animals and other organisms
☒	☐	Clinical data
☒	☐	Dual use research of concern
☒	☐	Plants

Methods

n/a	Involved in the study	
☒	☐	ChIP-seq
☒	☐	Flow cytometry
☒	☐	MRI-based neuroimaging

Plants

Seed stocks	*Report on the source of all seed stocks or other plant material used. If applicable, state the seed stock centre and catalogue number. If plant specimens were collected from the field, describe the collection location, date and sampling procedures.*
Novel plant genotypes	*Describe the methods by which all novel plant genotypes were produced. This includes those generated by transgenic approaches, gene editing, chemical/radiation-based mutagenesis and hybridization. For transgenic lines, describe the transformation method, the number of independent lines analyzed and the generation upon which experiments were performed. For gene-edited lines, describe the editor used, the endogenous sequence targeted for editing, the targeting guide RNA sequence (if applicable) and how the editor was applied.*
Authentication	*Describe any authentication procedures for each seed stock used or novel genotype generated. Describe any experiments used to assess the effect of a mutation and, where applicable, how potential secondary effects (e.g. second site T-DNA insertions, mosiacism, off-target gene editing) were examined.*
